# Development and characterization of a glycine biosensor system for fine-tuned metabolic regulation in *Escherichia coli*

**DOI:** 10.1186/s12934-022-01779-4

**Published:** 2022-04-07

**Authors:** Kun-Qiang Hong, Jing Zhang, Biao Jin, Tao Chen, Zhi-Wen Wang

**Affiliations:** 1 Department of Biochemical Engineering, School of Chemical Engineering and Technology, Tianjin University, Tianjin, 300072 People’s Republic of China; 2 Key Laboratory of Systems Bioengineering (Ministry of Education), Tianjin University, Tianjin, 300072 China; 3 SynBio Research Platform, Collaborative Innovation Center of Chemical Science and Engineering (Tianjin), Tianjin University, Tianjin, 300072 China; 4 Frontier Science Center for Synthetic Biology (Ministry of Education), Tianjin University, Tianjin, 300072 China

**Keywords:** Glycine riboswitch, Biosensor, *Escherichia coli*, Metabolic regulation, Directed evolution

## Abstract

**Background:**

In vivo biosensors have a wide range of applications, ranging from the detection of metabolites to the regulation of metabolic networks, providing versatile tools for synthetic biology and metabolic engineering. However, in view of the vast array of metabolite molecules, the existing number and performance of biosensors is far from sufficient, limiting their potential applications in metabolic engineering. Therefore, we developed the synthetic glycine-ON and -OFF riboswitches for metabolic regulation and directed evolution of enzyme in *Escherichia coli.*

**Results:**

The results showed that a synthetic glycine-OFF riboswitch (glyOFF6) and an increased-detection-range synthetic glycine-ON riboswitch (glyON14) were successfully screened from a library based on the *Bacillus subtilis* glycine riboswitch using fluorescence-activated cell sorting (FACS) and *tetA*-based dual genetic selection. The two synthetic glycine riboswitches were successfully used in tunable regulation of lactate synthesis, dynamic regulation of serine synthesis and directed evolution of alanine-glyoxylate aminotransferase in *Escherichia coli*, respectively. Mutants AGXT22 and AGXT26 of alanine-glyoxylate aminotransferase with an increase of 58% and 73% enzyme activity were obtained by using a high-throughput screening platform based on the synthetic glycine-OFF riboswitch, and successfully used to increase the 5-aminolevulinic acid yield of engineered *Escherichia coli*.

**Conclusions:**

A synthetic glycine-OFF riboswitch and an increased-detection-range synthetic glycine-ON riboswitch were successfully designed and screened. The developed riboswitches showed broad application in tunable regulation, dynamic regulation and directed evolution of enzyme in *E. coli*.

**Supplementary Information:**

The online version contains supplementary material available at 10.1186/s12934-022-01779-4.

## Background

Synthetic biology and metabolic engineering are pivotal tools in the design and construction of microbial cell factories for the biosynthesis of next-generation chemicals and materials. With the development of multiplex automated genome engineering (MAGE) [1], clustered regularly interspaced short palindromic repeats (CRISPR) [2, 3], and phage assisted mutagenesis editing technology [4], facile genome editing can be achieved in most microorganisms. CRISPR system has become the current mainstream gene editing method for its advantages in simplified operation, high editing efficiency and versatility. However, the problem of off-target effect should be addressed [5, 6]. Fine-tuned metabolic regulation is still essential for collaborative system optimization of metabolic networks to efficiently convert substrates into bioproducts [7, 8]. Deletion or constitutive expression of metabolic pathway genes in microbial cell factories under static control often leads to an increased metabolic burden and even growth retardation due to an imbalance of cofactors or the accumulation of toxic intermediates [9, 10]. Combinatorial libraries of promoters, RBSs (ribosome binding sites) and intergenic regions have been extensively studied as tools for the fine-tuned regulation of gene expression [11], but these strategies require efficient screening methods to select the most suitable gene expression levels from a large number of combinations. However, the outputs of these regulation systems are optimized for a particular set of conditions and are static, unable to respond to growth and environmental changes. By contrast, natural cells are capable of maintaining robust growth in the face of environmental fluctuations by dynamically adjusting the cellular metabolism through complex regulatory networks [12]. The fine-tuned regulation of gene expression is still a major bottleneck in the construction of microbial cell factories [13, 14]. Dynamic control systems or circuits will be important tools for the precise regulation of gene expression in collaborative system optimization of metabolic networks in microbial cell factories [15].

In a dynamic control system or circuit, a biosensor must be included as an essential element responsible for sensing the concentration of intracellular metabolites and transforming this information into a specific signal output. Extensive tunability, appropriate sensitivity, and a suitable threshold of biosensors would ensure the precise regulation of host metabolism. Biosensors and genetic control circuits have been successfully used in the dynamic regulation of pathways [7, 16, 17], enhancing the substrate utilization rate [8], as well as the product titer or productivity [18, 19], isolation and improvement of desired enzymes [20, 21], etc. Most in vivo biosensors were designed based on riboswitches [22, 23], transcription factors (TFs) [24, 25] and fluorescence resonance energy transfer (FRET) [26, 27]. Riboswitches are regulatory RNA segments located in the 5’-noncoding regions of mRNAs. Generally, a riboswitch consists of one or two aptamer domains for the binding of a small molecule that can be used as a signal for control, followed by an expression platform that mediates a conformational change, thereby affecting the initiation of transcription/translation, termination, or alternative splicing in response to the signal [28–30]. In 2002, Breaker et al. first discovered a metabolite-sensing riboswitch to control gene expression [31]. Since then, more than 40 different classes of riboswitches were found, responding to a diverse array of small molecules such as coenzymes, nucleotide derivatives, signaling molecules, ions, amino acids, and other metabolites [28]. Compared with TFs and FRET, riboswitches exhibited outstanding performance in terms of a short response time, reduced cellular metabolic burden and stricter regulation of gene expression [9, 32].

Natural amino acid riboswitches can be easily adapted to dynamically control metabolic pathways, and even be designed as a ‘synthetic switch’ to enhance the metabolic fluxes of the required metabolic pathways in engineered microorganisms. Amino acid riboswitches are of great significance for in vivo dynamic metabolic regulation, directed evolution and construction of microbial cell factories. To date, natural amino acid riboswitches for lysine [33–37], glycine [38–40], and glutamine [41–43] have been reported, and they were found to rely on elaborate and relatively abundant riboswitch aptamers. Although lysine and glutamine riboswitches have been used to expedite the evolution of microbes or enzymes and improve the yields of bioproducts [36, 37, 44, 45], the relative contribution of each aptamer toward gene regulation and the structural basis of riboswitch function are still controversial [40, 46]. Recently, a new amino acid riboswitch that responds to tryptophan was also used to increase Trp production by improving the product tolerance of *Escherichia coli* [47], but the number of currently available biosensors is still limited in comparison with the vast number of metabolite molecules, limiting their potential applications in microbial cell factories.

In 2004, a glycine riboswitch was first discovered using cooperative binding to control gene expression [48], after which similar riboswitches were widely identified in both Gram-positive and -negative bacteria, such as *Bacillus subtilis* [48], *Streptococcus pyogenes* [49], *Vibrio cholera* [50], *Fusobacterium nucleatum* [51], and *Clostridium pasteurianum* [52]. In vitro experimental approaches of glycine riboswitch research provided valuable insights into the structure and mechanism of action of these riboswitches [40, 53, 54], but in vivo experiments more closely reflect their behavior in a complex metabolic environment and have great potential in metabolic engineering. Glycine riboswitches from *B. subtilis* and *S. pyogenes* were successfully used for the controllable overproduction of recombinant proteins [49, 55], and examination of its function in a biologically relevant context in native cells [56]. An in vivo glycine riboswitch was used for the facile control of recombinant protein production in native cells [49, 55, 56], but there is only one report of using a glycine riboswitch for controlling an indirectly related metabolic pathway for a biosynthesis purpose [52]. A synthetic glycine-OFF riboswitch was screened from a *C. pasteurianum* glycine-ON riboswitch library and successfully used to downregulate the expression of the *hemB* gene (encoding porphobilinogen synthase, which uses 5-ALA), resulting in a significant increase (11%) of the 5-aminolevulinic acid titer [52]. Glycine riboswitches have been used to regulate gene expression by coupling glycine binding to a structural transition of the riboswitch. However, more attention should be still paid to understanding the relationships between the sequence or structure and regulatory function, improving riboswitch performance, developing more available riboswitches for metabolic engineering.

In this study, glycine riboswitches from four different sources were selected to characterize their performance in *E. coli*. By combining *tetA*-based dual genetic selection and screening via fluorescence-activated cell sorting (FACS), we successfully obtained a synthetic glycine-OFF riboswitch and an increased-detection-range synthetic glycine-ON riboswitch from a library of the *B. subtilis* glycine riboswitch. The two synthetic glycine riboswitches were successfully used in tunable regulation, dynamic regulation and directed evolution of enzymes in *E. coli*. Some novel mutation sites were enriched, which might have a positive effect on changing the conformation and improving the performance of the riboswitch. Mutants of alanine-glyoxylate aminotransferase with improved enzyme activity were screened using a high-throughput screening platform based on the synthetic glycine-OFF riboswitch, and successfully used to increase the 5-ALA yield of engineered *E. coli*. This work provides new synthetic glycine-ON and -OFF riboswitches for metabolic engineering, and presents a valuable example of modifying a wild-type riboswitch from physiological to industrial significance.

## Results

### Selection and construction of an in vivo glycine riboswitch suitable for *E. coli*

**Fig. 1:**
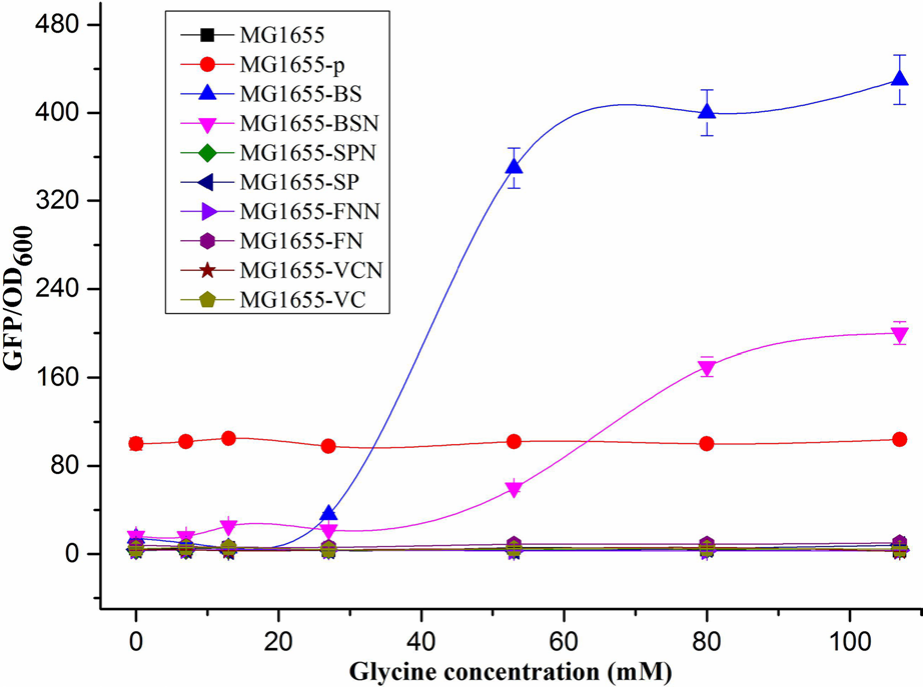
Relative GFP expression (GFP/OD_600_) of different glycine riboswithches expressed in MG1655 strain. The relative GFP expressions were measured in M9 medium supplemented with 0, 7, 13, 27, 53, 80, and 107 mM glycine, and MG1655 was cultured in M9 medium without ampicillin. All data are the average values of three independent experiments

To find a suitable in vivo glycine riboswitch for *E. coli,* four reported in vitro glycine riboswitches from *B. subtilis*, *S. pyogenes*, *V. cholerae*, and *F. nucleatum* were screened with GFP as a reporter in the high copy number plasmid pUC18 under the native promoter or synthetic M1-37 promoter [57], respectively. The growth performance of the strains was significantly negatively affected when the glycine concentration exceeded 107 mM (Additional file 1: Fig. S1). Excess glycine and the accumulation of ammonia and methylene tetrahydrofolate produced by glycine cleavage (GCV) enzyme system are toxic to host cell [58]. Therefore, the highest concentration of glycine in the medium was less than 107 mM in this study. The eight glycine riboswitch plasmids and the control plasmid were characterized in *E. coli* MG1655, which were cultured in M9 medium with various concentrations of glycine (0, 7, 13, 27, 53, 80, 107 mM). The dose–response of the glycine riboswitches, assessed by measuring the fluorescent protein intensity (Fig. 1). Unexpectedly, only the glycine riboswitch from *B. subtilis* functioned as an activator to promote the downstream *gfp* gene expression. Riboswitch function relies on a balance between multiple factors including the transcription rate, ligand binding kinetics and RNA folding [59, 60]. The other glycine riboswitches not exhibited their function in *E. coli* might be due to the greater differences of genetic background or organism’s native environment than that between *B. subtilis* and *E. coli*. In addition, the native promoter of the riboswitch might be a good choice if it is compatible with the bacterial species. Alternatively, a well-characterized constitutive promoter would be a better choice if the native promoter is incompatible [59]. The synthetic promoter M1-37 [57] was found to be more compatible than the native promoter in our work, resulting in *B. subtilis* glycine riboswitch controlled by synthetic promoter M1-37 in MG1655-BS exhibited more significant dynamic regulation than MG1655-BSN with native promoter (Fig. 1 and Additional file 1: . S2).

*tetA* gene has the unique characteristic of being able to function as both positive selection marker for retaining tetracycline resistance and negative selection marker for losing sensitivity to chemicals such as NiCl_2_ [61]. To construct the dual genetic selection cassette, the *tetA* gene was fused with the *gfp* gene under the control of the *B. subtilis* glycine-ON riboswitch (Additional file 1: Fig. S3a). When the reporter gene *tetA* was expressed in the presence of glycine, the host cell become resistant to tetracycline and sensitive to NiCl_2_ with the accumulation of TetA. Conversely, attenuation of *tetA* expression in the absence of glycine resulted in a NiCl_2_ insensitive and tetracycline in-resistant phenotype. The GFP/OD_600_ value of MG1655-BSTG was consistent with that of MG1655-BS under the same conditions, indicating that the insertion of the *tetA* gene did not affect glycine riboswitch function (Additional file 1: Fig. S3b). The growth rate (μ) of MG1655-BSTG decreased gradually with the increase of exogenous Ni^2+^ concentration in M9 medium supplemented with 80 mM glycine (Additional file 1: Fig. S3c). The μ value of MG1655-BSTG in the presence of 90 μM Ni^2+^ was 0.045 h^−1^, approximately fivefold lower than in the absence of Ni^2+^(Additional file 1: Fig. S3c). Excessive Ni^2+^ almost completely inhibited the growth of the strain MG1655-BSTG. The growth performance of MG1655-BSTG was also characterized in M9 medium supplemented with 90 μM Ni^2+^ and different concentrations of glycine. The maximum OD_600_ value of MG1655-BSTG in the presence of 80 mM glycine was sixfold lower than in the absence of glycine (Additional file 1: Fig. S3d). The *tetA*-based dual genetic selection system was therefore successfully constructed using the *B. subtilis* glycine riboswitch, and could be further used for the screening of the glycine riboswitch library.

### Screening and characterization of glycine-ON and -OFF libraries

**Fig. 2:**
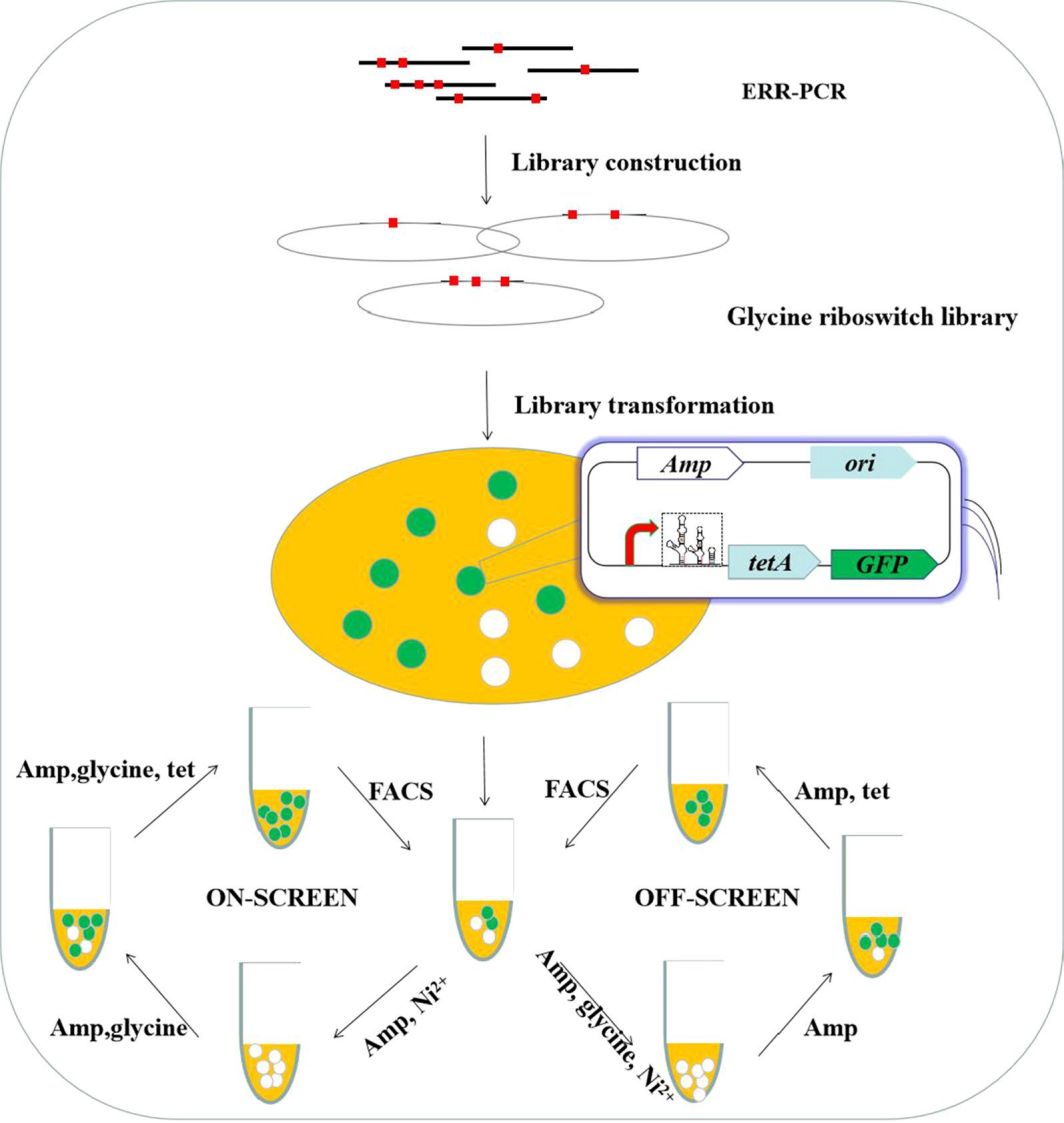
Screening workflow of synthetic glycine-ON and -OFF riboswitches

To develop synthetic glycine-ON and -OFF riboswitches, the glycine riboswitch library and backbone fragment were assembled together via Gibson assembly and transformed into *E. coli* MG1655 competent cell after four rounds of error-prone PCR. Glycine-ON plasmid libraries were screened with the assay of ‘ON-SCREEN’ and glycine-OFF plasmid libraries were screened according to the assay of ‘OFF-SCREEN’ (Fig. 2). After three rounds of in vivo dual selection, ~ 1.5 × 10^4^ cells with the highest fluorescence were sorted by FACS (fluorescence-activated cell sorting) from a total of ~ 2.5 × 10^7^ cells harboring glycine-ON riboswitches. Similarly, ~ 10^4^ cells with the highest fluorescence were sorted by FACS from a total of ~ 10^7^ cells harboring glycine-OFF riboswitches.

**Fig. 3:**
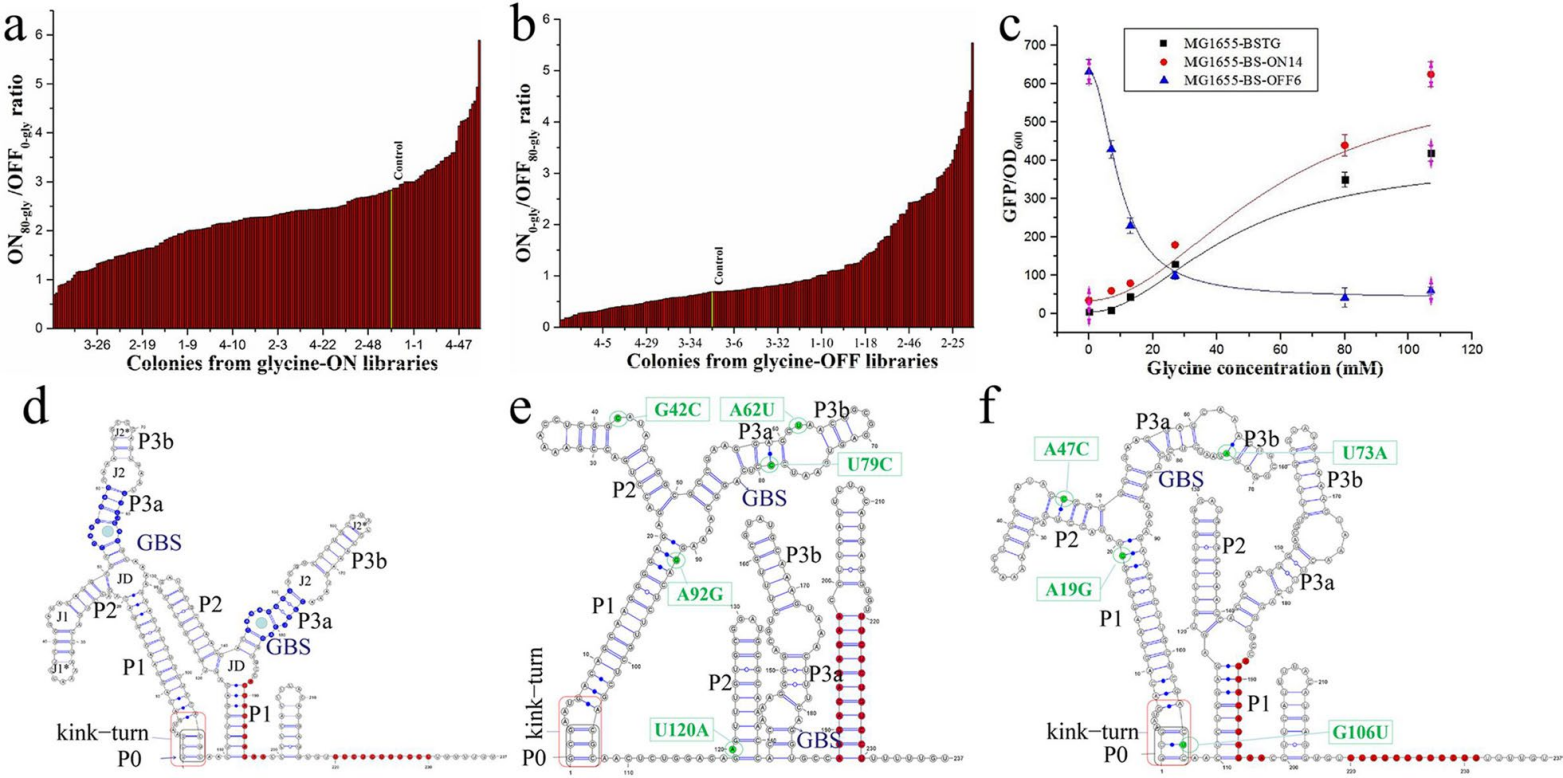
Selection and characterization of glycine-ON and -OFF switches from mutant libraries. **a** ON/OFF ratio of relative fluorescence intensity in the wild-type glycine riboswitch and the selected glycine-ON riboswitches in M9 medium with or without 80 mM glycine in 96-deep-well plates. **b** ON/OFF ratio of relative fluorescence intensity in the wild-type glycine riboswitch and the selected glycine-OFF riboswitches in M9 with or without  80 mM glycine in 96-deep-well plates. The yellow column indicates the control strain. **c** GFP/OD_600_ ratio of MG1655-BSTG, G1655-BS-ON14, and MG1655-BS-OFF6. Strains were cultured in M9 medium supplemented with 0, 7, 13, 27, 80, and 107 mM glycine. All the data are the average values of three independent experiments. **d** Secondary structure of the wild-type *B. subtilis* glycine riboswitch. **e** Mutations in the glycine-OFF riboswitch glyOFF6 affecting the secondary structure. **f** Mutations in the glycine-ON riboswitch glyON14 affecting the secondary structure. Base-pairing stems were labeled as the P0/kink-turn motif, P1, P2, and P3 with subsections labeled ‘a’ and ‘b’, and junction structures were labeled J1, J2 and JD with subsections labeled ‘*’. Mutant sites were highlighted in green nucleotides and green boxes with mutation information, red shading highlights nucleotides that base pair to form the transcription terminator stem when the riboswitch is in the ‘OFF’ conformation. Riboswitches’ structures and its mutations were analyzed by RNAflod and VARNA 3–93

The top 0.05–0.1% of cells with the highest fluorescence were allowed to regenerate in SOC medium for 50 min and cultured on LB agar plates with or without 80 mM glycine and 90 μM NiCl_2_. About 200 single colonies containing presumed glycine-ON riboswitches and 200 single colonies with presumed glycine-OFF riboswitches were randomly picked and cultured in 96-deep-well plates containing 1 mL of M9 medium with or without 80 mM glycine, and the GFP/OD_600_ value of the was measured, respectively. A total of 32 mutants from the glycine-ON library exhibited higher ON/OFF values than the control (Fig. 3a), and 118 mutants from the glycine-OFF library exhibited significant repression compared with the wild type (Fig. 3b). Subsequently, glyON14 and glyOFF6 with the highest ON/OFF ratios were rescreened in 50 mL of M9 medium with various concentrations of glycine. As shown in Fig. 3c, mutant riboswitch glyOFF6 showed significant repression in the presence of glycine, and activation in the absence of glycine. glyOFF6 shown over tenfold repression compared with that of glyOFF6 in the presence of 107 mM glycine, and 54% higher than the response amplitude of the wild-type riboswitch. Similarly, the GFP/OD_600_ value of glyON14 was increased by 50% in the presence of 107 mM glycine. Additionally, the detection range was increased to 107 mM from 80 mM. The *K*_*d*_ value of glyON14 for glycine was increased to 49 mM, 1.53-fold higher than that of the wild-type glycine riboswitch (Additional file 1: Table S7), indicating that the synthetic glycine-ON riboswitch had lower sensitivity for glycine. A synthetic glycine-ON riboswitch (glyON14) with wider detection range and a synthetic glycine-OFF riboswitch (glyOFF6) were successfully screened from the glycine riboswitch libraries using *tetA*-based dual genetic selection and FACS selection.

### Sequence analysis of the evolved glycine riboswitches

To dissect these contributions, the top six synthetic glycine-ON riboswitches and top six synthetic glycine-OFF riboswitches (Additional file 1: Table S8) with the highest ON/OFF value, including glyON14 and glyOFF6, were sequenced to explore the effect of mutations on the function of synthetic glycine riboswitches.

Glycine riboswitch mutant glyOFF6 contained five novel mutations, G42C, A62U, U79C, A92G, and U120A (Fig. 3d, e). The transcription terminator stem of glyOFF6 was formed after glycine binding, resulting in the ‘OFF’ conformation, while these base pairs were free in the ‘ON’ conformation in the absence of glycine (Fig. 3e). The G42C and A62U mutations were positioned in junction structures J1 and J2, respectively. U79C was positioned in adjacent to the glycine binding site (GBS) in the first aptamer, while A92G and U120A were positioned in the P1 and P2 helices, respectively (Fig. 3e). Interestingly, two additional synthetic glycine-OFF riboswitches also included mutations in the GBS, A56G in glyOFF5, as well as C143A, U177C, and U179A in glyOFF11 (Additional file 1: Table S9). We also observed that the high frequency mutation U120A was found in glyOFF6, glyOFF8 and glyOFF35 (Additional file 1: Table S9), which might alter the wild-type glycine-ON riboswitch into glycine-OFF riboswitch. Another high frequency mutation site was positioned in the linker sequence, A108G in glyOFF5 and A108U in glyOFF12 (Additional file 1: Table S9), which might affect the cooperative mode of the two aptamers. These eight novel mutations (A56G, U79C, C143A, U177C, U179A U120A, A108G, and A108U) might alter the wild-type conformation. Furthermore, the proportions of mutations occurring in the second aptamer of the six glycine-OFF riboswitches were higher than in the six glycine-ON riboswitches (Additional file 1: Fig. S4), which was consistent with the previous report of tandem glycine-OFF riboswitch variants were shown to exhibit preferential binding to the second aptamer [40].

Another increased-detection-range and increased-amplitude-value synthetic glycine-ON riboswitch, glyON14, contained the four novel mutations A19G, A47C, U73A, and C106U (Fig. 3d, f). A19G and A47C were respectively positioned in the P1 and P2 helices, U73A was positioned in the junction structure subsection J2*, and C106U was located in the P0/K-turn (‘P0’ helices and kink-turn motif) (Fig. 3f). The A19G mutation located in the P1 helices and other mutations located in the P3A or P3B helices (Additional file 1: Fig. S5). The mutations located in the P1 helices would change the adenosine located in the glycine-binding pocket to form a δ interaction and thus effect glycine binding in both aptamers [30]. Glycine riboswitches containing the P0/K-turn would affect glycine binding affinities and cooperation [50, 53, 62]. The mutations A4C in GLYON4, A5G and A8U in GLYON13, as well as C106U in GLYON14 were positioned in the P0/K-turn, which should influence the glycine binding affinity and thus increase the detection range. In addition, the mutation U179A was enriched in both the synthetic glycine-ON and -OFF libraries (Additional file 1: Table S9), and U179 was previously reported as a key site influencing the amplitude value in in vitro experiments [40].

### Tunable regulation of lactate synthesis in *E. coli* using a glycine-ON riboswitch

**Fig. 4:**
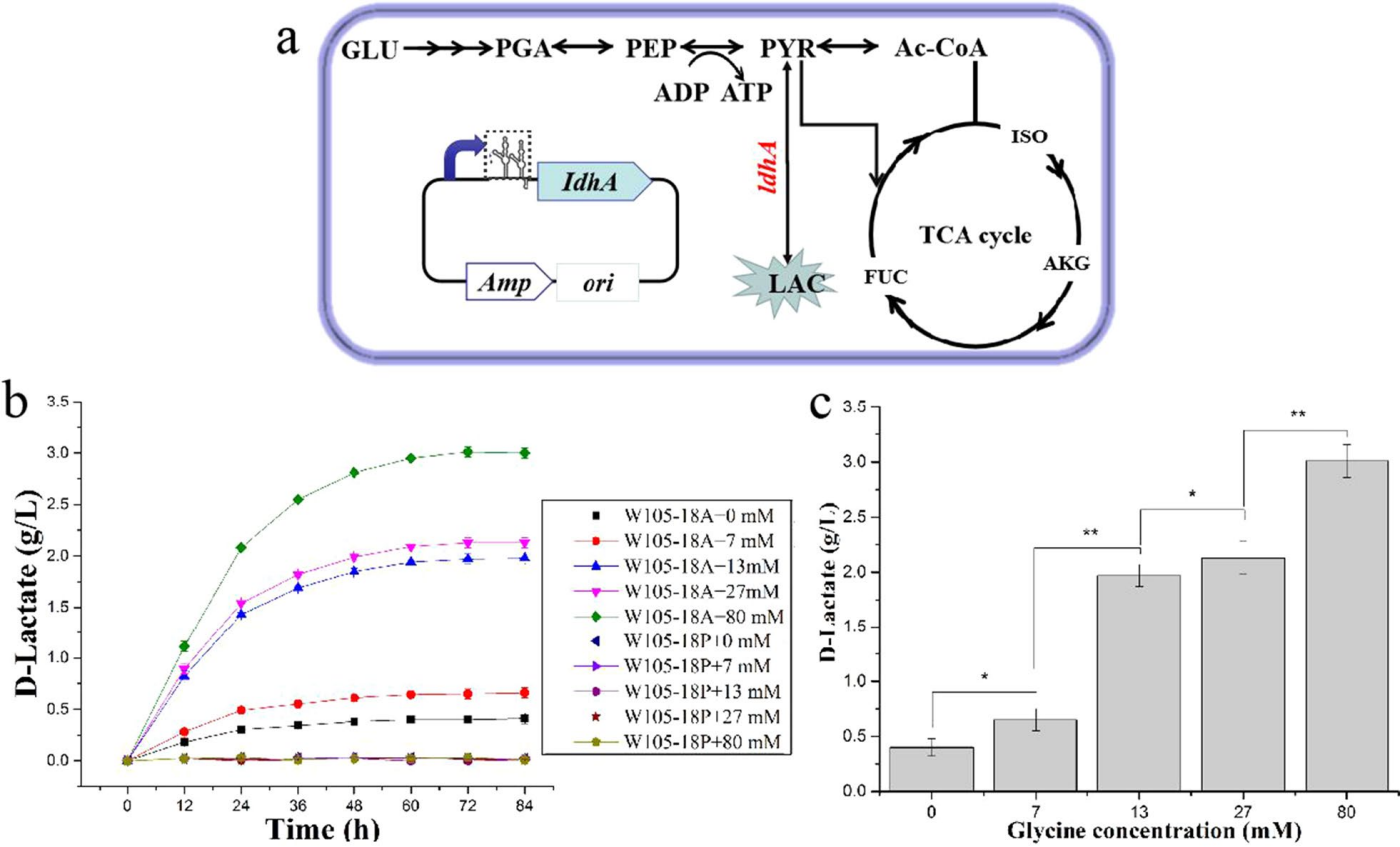
Tunable regulation of lactate synthesis using the evolved glycine-ON riboswitch glyON14 in *E. coli.* **a** Schematic representation of the metabolic pathways involved in lactate production from glucose in *E. coli*. The expression of *ldhA,* encoding lactate dehydrogenase, was dynamically controlled under the synthetic glycine-ON riboswitch glyON14 in the high-copy-number plasmid pUC18. GLU, glucose; PGA, 3-phosphoglycerate; PEP, phosphoenolpyruvate; ISO, isocitrate; AKG, α-ketoglutaric acid; FUM, fumaric acid; TCA cycle: tricarboxylic acid cycle; PYR, pyruvate; LAC, lactate; Ac-CoA, acetyl coenzyme A; Amp, ampicillin; r, resistance; glycine-ON riboswitch glyON14 in the dashed line box; ori, replicon. **b** Lactate production of engineered strain W105-18A and the control strain W105-18P in M9 medium supplemented with 0, 7, 13, 27, and 80 mM glycine. **c** Maximal lactate concentration of W105-18A and W105-15A in M9 medium supplemented with 0, 7, 13, 27, and 80 mM glycine. Error bars represent the standard deviations of triplicate samples.**P < 0.01; *P < 0.05

To verify the usability of the synthetic glycine-ON riboswitch in metabolic engineering, glyON14 was used for tunable regulation of the *ldhA* gene (encoding lactate dehydrogenase) in the evolved lactate-producing *E. coli* strain W105 (Fig. 4a). glyON14 was fused with the *ldhA* gene in high-copy-number plasmid pUC18, 15-copy-number plasmid P15C and lower 5-copy-number plasmid pZY48, respectively. The concentration of lactate in W105-18A harboring pUC18-GlyON-*ldh*A plasmid increased more obviously with the increase of exogenous glycine (Fig. 4b, c). The strains W105-15A and W105-48A harboring the low-copy-number plasmids P15C-GlyON-ldhA and Pzy48-GlyON-ldhA produced higher concentration of lactate, 4.51 and 4.42 g L^−1^, respectively (Additional file 1: Fig. S7). However, the concentrations of lactate in the strains harboring the low-copy-number plasmid P15C-GlyON-ldhA and pZY48-GlyON-ldhA were not significantly increased when the concentration of exogenous added glycine was over 7 mM (Additional file 1: Figs. S7, S9a, and S10a). W105-18P, W105-15P and W105-48P harboring the control plasmids not produced lactate with or without glycine (Fig. 4b, Additional file 1: Figs. S9a and S10a). The growth of W105-18A was gradually decreased with the increase of exogenous glycine compared with that of W105-15A and W105-48A harboring the low-copy-number plasmids and their control strains (Additional file 1: Fig S6a, Fig S9b and S10b). Therefore, glycine riboswitch integrated into the high-copy-number plasmids had a better gradient response, as consistent with the previous report of a high copy number will help increase the strength of the signal [60]. The saturation phenomenon in W105-15A and W105-48A maybe due to the low copy number of the expression plasmids, which make the glycine riboswitch insensitive to the glycine concentration. The strain harboring the high-copy-number plasmid produced abundant foreign proteins, resulting in a metabolic burden for the cells and thus decreasing the cell growth [63], which could explain the metabolite and growth changes of these strains (Additional file 1: Fig S6, S9 and S10). To further validate the usability of glyON14 in metabolic engineering, lactate fermentation of W105-15A harboring P15C-GlyON-ldhA was performed under lower than 7 mM glycine. The maximal concentration of lactate produced by strain harboring low-copy-number plasmid also gradually increased in the appropriate range (Additional file 1: Fig S8).

### Dynamic metabolic regulation using the glycine-OFF riboswitch in *E. coli*

**Fig. 5:**
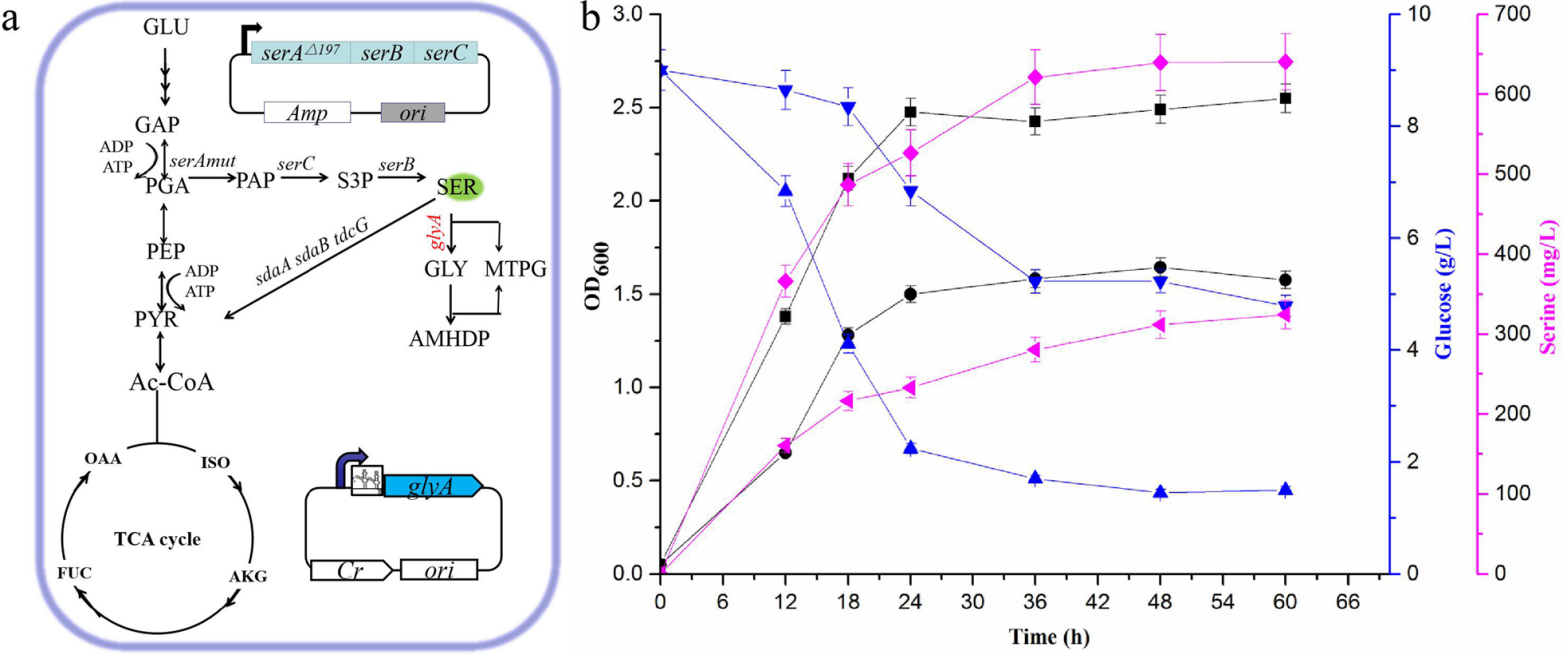
Dynamic metabolic regulation for improved serine production in *E. coli* using the glycine-OFF riboswitch glyOFF6. **a** Schematic representation of the metabolic pathways involved in serine production from glucose in recombinant *E. coli*. The expression of *glyA*, encoding serine hydroxymethyltransferase, was dynamically regulated under the synthetic glycine-OFF riboswitch glyOFF6 in plasmid pUC18, co-expressed with pPK10 to produce serine in *E. coli.* The genes *sdaA*, encoding L-serine deaminase I; *sdaB*, encoding L-serine deaminase II; and *tdcG*, encoding L-serine deaminase III were deleted, *serA*^*Δ197*^, (encoding a truncated 3-phosphoglycerate dehydrogenase gene from *C. glutamicum*), *serC* (encoding phosphoserine aminotransferase), *serB* (encoding phosphoserine phosphatase) and *pgk* (encoding phosphoglycerate kinase) were upregulated by overexpression using the plasmid pPK10. GLU, glucose; PGA, 3-phosphoglycerate; PEP, phosphoenolpyruvate; ISO, isocitrate; AKG, α-ketoglutaric acid; FUM, fumaric acid; OAA, oxaloacetic acid; TCA cycle: tricarboxylic acid cycle; PGA, 3-phosphoglycerate; PEP, phosphoenolpyruvate; PYR, pyruvate; Ac-CoA, acetyl coenzyme A; PAP, 3-phosphopyruvate; S3P, 3-phosphoserine; MTPG, methylene-tetrahydropteroyl polyglutamate; AMHDP, s-amino-methyldihydrolipoyl protein; GLY, glycine; SER, serine; Amp: ampicillin, Cr: chloramphenicol resistance gene; ori, replicon; glycine-OFF riboswitch glyOFF6 in the solid line box; **b** OD_600_, glucose consumption, and serine production of engineered strain E4GS and control strain E4GD in M9Y medium under aerobic conditions with an initial OD_600_ of 0.05. (Black solid circle: E4GD OD_600_; Black solid square: E4GD OD_600_; Blue falling triangle: E4GD glucose consumption; Blue positive triangle: E4GS glucose consumption; Red diagonal triangle: E4GD serine production; Red rhombus: E4GS serine production) Error bars represent the standard deviations of triplicate samples

*glyA* gene, encoding L-serine hydroxymethyltransferase, catalyzes the conversion of serine into glycine. Deletion of the *glyA* gene would lead to growth arrest in minimal medium, and its inducible expression might increase the production costs. To dynamically regulate *glyA* gene expression using the glycine-OFF riboswitch in *E. coli*, glyOFF6 was fused with the *glyA* gene in pZY48 to control serine synthesis in a serine-producing strain E4G2, in which *glyA* gene was deleted (Fig. 5a). As shown in Fig. 5b, the control strain E4GD (E4G2 harboring pZY48) consumed 4.2 g L^−1^ glucose and produced 324.49 mg L^−1^ serine with a yield of 77.26 mg g^−1^ glucose, reaching a maximal OD_600_ of 1.58 in 60 h. By contrast, the E4GS strain (E4G2 harboring pZY48-glyOFF6-glyA, *glyA* gene was regulated by glyOFF6) exhibited a 104% increase of serine production (660.48 mg L^−1^) with a yield of 85.40 mg g^−1^ glucose. Moreover, the growth performance of E4GS was also improved, with a maximal OD_600_ of 2.55 (Fig. 5b). Hence, the expression of the *glyA* gene was dynamically controlled by the glycine-OFF riboswitch, realizing efficient resource utilization and an improvement of serine productivity, while also minimizing the need for chemical inducers. These results confirmed that it is possible to precisely regulate the expression of a key gene in an important metabolic pathway using our synthetic glycine-OFF riboswitch in *E. coli*.

### Directed evolution of alanine-glyoxylate aminotransferase using a synthetic high-throughput screening platform based on the glycine-OFF riboswitch

**Fig. 6:**
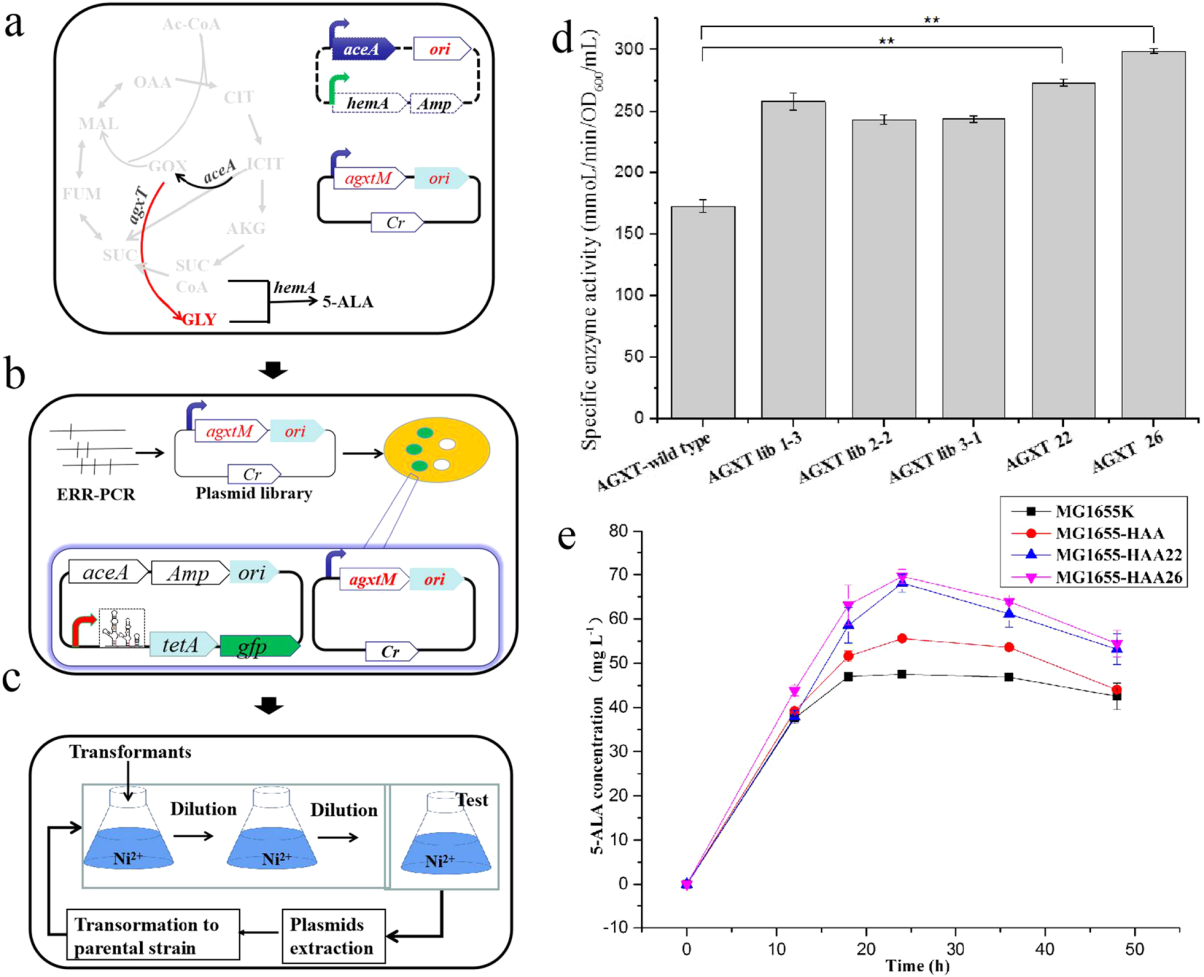
Synthetic glycine-OFF riboswitch used for the directed evolution of alanine-glyoxylate aminotransferase in *E. coli.* **a** A mutant of AGXT with higher enzyme activity was screened using a high-throughput screening platform based on the synthetic glycine-OFF riboswitch. The genes *agxtM*, *aceA* and *hemA* were co-expressed from two plasmids to syntheze 5-ALA in *E. coli.* The glyoxylate pathway is highlighted in red. **b** The library of *agxT* mutants was constructed using error-prone PCR (Additional file 1: Table S6). The library plasmids and puc18-GlyOFF6-aceA plasmid were co-expressed in *E. coli* MG1655*.* **c** All clones from LB plates were transferred to M9 medium, and selected in fresh M9 medium supplemented with 100 μg mL^−1^ ampicillin and 90 μM Ni^2+^ to remove the strains with lower levels of AGXT enzyme activity. AGXT mutants with higher activity were enriched in three cycles, and the plasmids from each cycle were extracted and transferred to the original strain. The percentage of beneficial mutations and enzyme activity were also determined. The relevant genes include *aceA*, encoding isocitrate lyase; *agxTM*, encoding mutant glyoxylate transaminase; *hemA*, encoding 5-aminolevulinic acid synthase; *tetA*, tetracycline resistance gene; *gfp*, encoding green fluorescent protein. Ac-CoA, acetyl coenzyme A; CIT, citrate; ICIT, isocitrate; GOX, glyoxylate; AKG, α-ketoglutaric acid; SUCCoA, succinyl-CoA; SUC, succinic acid; FUM, fumaric acid; MAL, malic acid; OAA, oxaloacetic acid; Gly, glycine; 5-ALA, 5-aminolevulinic acid; Amp: ampicillin; Cr: chloramphenicol; ori, replicon. **d** Specific enzyme activity of wild-type AGXT, three library strains, AGXT22, and AGXT26. **e** 5-aminolevulinic acid concentrations of MG1655K, MG1655-HAA, MG1655-HAA22, and MG1655-HAA26. All the data are the average values of three independent experiments.**P < 0.01

Glycine biosensres sense and respond to glycine, which is produced from glyoxylate by AGXT (alanine-glyoxylate aminotransferase, encoded by *agxT*) (Fig. 6a). Glyoxylate pathway was engineered to produce glycine by expressing *aceA* (encoding isocitrate lyase) and *agxT* from two plasmids (Fig. 6a). ~ 10^4^ colonies on the agar plates were used for plasmid isolation to prepare the *agxT* library. 20 randomly picked clones all contained mutation sites revealing the expected mutations of *agxT* gene. The plasmids encoding the *agxT* library were co-electroporated with pUC18-BSTGOFF6-aceA into *E. coli* MG1655, and the transformants were cultivated in the presence of 90 μM NiCl_2_ (Fig. 6b). The strains harboring beneficial AGXT mutants with higher activities would produce higher concentration of glycine, resulting in TetA was inactivated, which would render the host cell insensitive to NiCl_2_. Strains harboring higher AGXT enzyme activity would be enriched by gradually suppressing the *tetA* selection marker necessary for survival in M9 medium supplemented with 90 μΜ Ni^2+^, and the surviving strains could produce higher levels of glycine (Fig. 6b, c). Beneficial mutations gradually accumulated after three selection cycles (Additional file 1: Fig. S9a). The top three mutations reached about 85, 90, and 100% of the total population by the third cycle. The growth rate of the strain in the third-round of cultivation was measured (Additional file 1: Fig. S9b). AGXT-lib2 has the highest growth rate of 0.045 h^−1^ (Additional file 1: Fig. S9b). These results indicate that this synthetic high-throughput screening platform is capable of enriching beneficial mutated plasmids under selective conditions due to the different growth rates of the high- and low-glycine producers. A total of 23 improved *agxT* variants from the third enrichment cycle were reconstructed in the pZY48 plasmid, and then the alanine-glyoxylate aminotransferase activity of these mutants was measured, along with three *agxT* library strains. The enzyme activities of the reconstructed mutants and strains from three libraries were all higher than that of wild-type alanine-glyoxylate aminotransferase (Additional file 1: Fig. S10). These results can be attributed to the fact that strains with higher AGXT enzyme activity and glycine production were enriched under these selection conditions, while the neutral and negative mutations produced by error-prone PCR were removed by the screening platform. The highest activities of the mutants AGXT22 and AGX2T6 were 273.37 and 298.98 (mmoL min^−1^OD_600_^−1^ mL^−1^), which was respectively 58 and 73% higher than that of wild-type AGXT (Fig. 6d). Next, shake-flask fermentations for 5-ALA production were performed using MG1655HAA, MG1655HAA22, and MG1655HAA26, respectively harboring the control and the two mutants. As shown in Fig. 6e, the maximal concentrations of 5-ALA produced by MG1655HAA22 and MG1655HAA26 were 64.56 and 69.64 mg L^−1^, representing 22.6 and 25.3% increases over MG1655HAA. Clearly, the glycine-OFF riboswitch-based high-throughput screening platform was successfully used for the directed evolution of alanine-glyoxylate aminotransferase in *E. coli*.

### Characterization of the evolved alanine-glyoxylate aminotransferase AGXT26

**Fig. 7:**
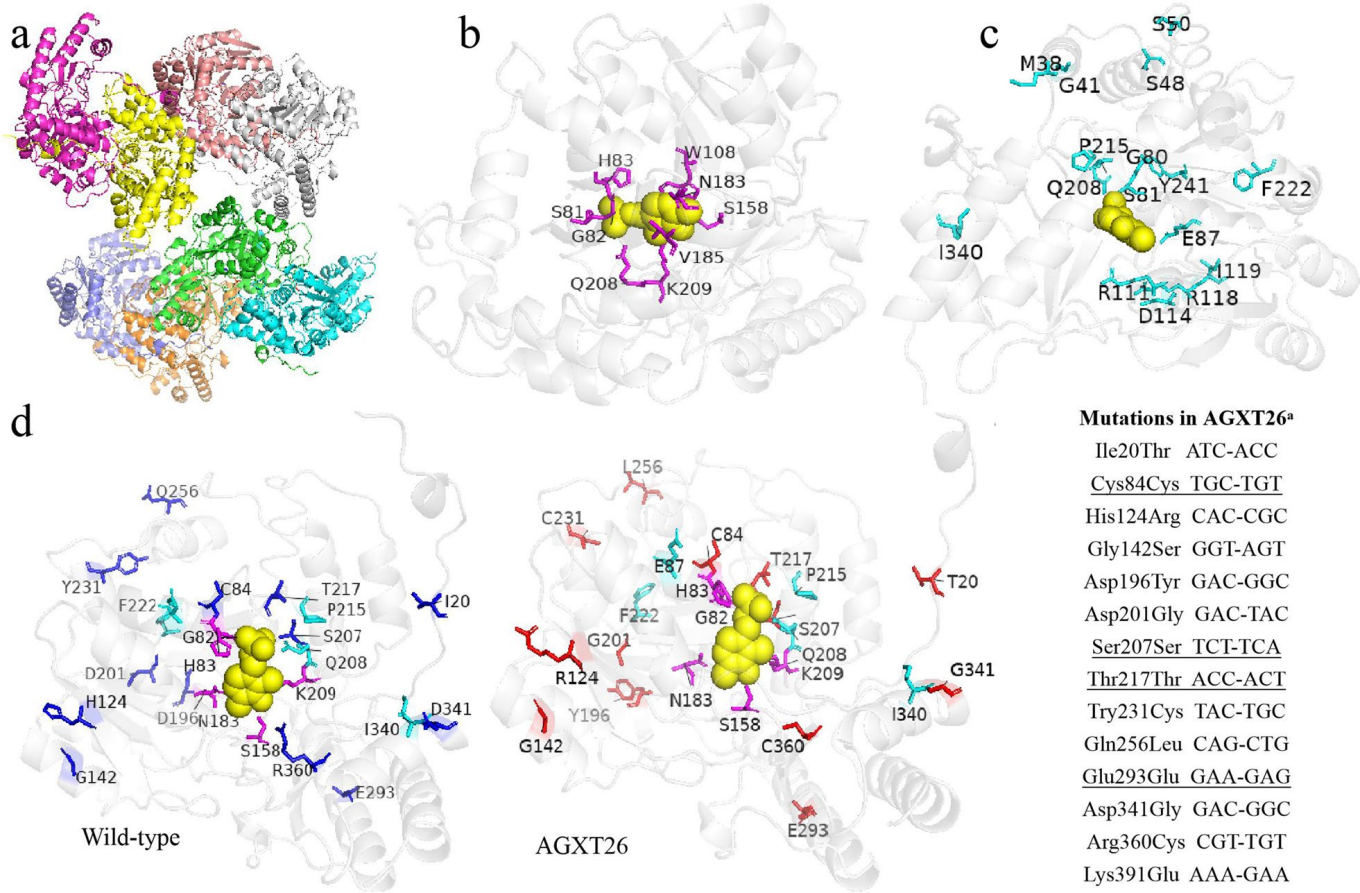
Crystal structures of wild-type AGXT and mutants. **a** PLP binding residues in a single subunit. K209 is both a catalytic residue and one of the PLP-binding residues. The catalytic lysine and PLP-binding residues are shown as magenta sticks. **b** Polypeptide binding sites of a single subunit. The polypeptide binding residues are shown as cyan sticks. **c** Mutation sites in AGXT26. The 13 wild-type residues are marked on the left, and the corresponding mutant residues in AGXT26 are marked on the right. Wild-type residues were shown as blue sticks, the mutant residues are shown as red sticks; PLP (pyridoxal-5’-phosphate) is shown in yellow. The image was rendered using PyMOL. **d** Details of the mutation sites in AGXT26. ^a^ Synonymous mutation are underlined

The alanine-glyoxylate aminotransferase from *Homo sapiens* was used as the starting enzyme for directed evolution in this work. AGXT (PDB ID:5LUC) is a homodimeric pyridoxal 5’-phosphate (PLP) dependent enzyme that catalyzes the interconversion of alanine and glyoxylate into pyruvate and glycine [64]. One homodimeric protein contains eight subunits (Fig. 7a), each of which has a catalytic K209 residue, nine PLP-binding residues (Fig. 7b), and twenty-one polypeptide binding sites (Fig. 7c). The crystal structure of *Homo sapiens* AGXT (PDB ID:5LUC) was used as modeling template to show the locations of mutations. AGXT26 included 14 mutations (Fig. 7d), of which Arg360Cys was near residue 158 in the PLP binding site, the Asp201Gly and Asp341Gly mutations of AGXT26, as well as the Met38Thr**,** Lys51Arg and Arg241Ala mutations of AGXT22 (Additional file 1: Fig. S11) were positioned in polypeptide binding sites. In addition, the residues 1 to 21 of the N-terminal extension are wrapped over the surface by interacting with residues 42–56 of another subunit of alanine-glyoxylate aminotransferase [65]. The mutation Ile20Thr of AGXT26 changed the distance between two subunits through the interaction between subunits (Additional file 1: Fig S12). The distance between the mutation site 196 and the 7th residue of another subunit of AGXT26 was closer than that of the wild-type enzyme (Additional file 1: Fig. S13), which might make the mutant more stable. Furthermore, four synonymous mutations (84th, 207th, 217th, 293rd) were also found in AGXT26 (Fig. 7d). According to the Codon Usage Database (http://www.kazusa.or.jp/codon/), a higher-frequency codon (UCA) was introduced into the synonymous mutation at 207th site (Fig. 7d).

## Discussions

Static control of metabolic pathway genes in microbial cell factories often leads to growth arrest or metabolic burden [10, 11, 36, 66]. Combinatorial libraries have been extensively developed for regulation of metabolic pathway genes, but these strategies are unable to respond to growth environmental changes and also require efficient screening methods from a large number of combinations [11]. In recent years, biosensors have been increasingly used in metabolic engineering and synthetic biology, they can be implemented in synthetic circuits to control the expression of key genes in response to specific cellular stimuli [9, 10, 66], and maintain robust growth in the face of environmental fluctuations [12]. Numerous biosensors including riboswitches, TFs and FRET were reported [28], however, the number of currently available biosensors is still limited in comparison with the vast number of metabolite molecules. There is few report of using a glycine riboswitch for controlling an indirectly related metabolic pathway for a biosynthesis purpose [52]. More attention should be paid to improving riboswitch performance or developing more available riboswitches for metabolic engineering. This work focused on development and characterization of synthetic glycine riboswitches for metabolic regulation in *E. coli*. A synthetic glycine-OFF riboswitch (glyOFF6) with significant repression in the presence of glycine and an increased-detection-range (0–107 mM) glycine-ON riboswitch (glyON14) were developed from a library based on the *B. subtilis* glycine riboswitch using fluorescence-activated cell sorting (FACS) and *tetA*-based dual genetic selection. The two synthetic glycine riboswitches were implemented in synthetic circuits and successfully used in tunable regulation of lactate synthesis, dynamic regulation of serine synthesis and directed evolution of alanine-glyoxylate aminotransferase in *E. coli*, respectively.

A biosensor-based dynamic regulation system can sense the metabolite level and convert the input into a transcriptional signal to dynamic regulate a key pathway gene [67]. L-serine hydroxymethyltransferase (SMHT), encoded by the *glyA* gene, catalyzes the conversion of serine into glycine (Fig. 5a). To increase L-serine production, *glyA* gene was usually knocked out directly to prevent L-serine degradation [68], as well as SMHT activity was maintained at a low level by blocking the folate biosynthesis pathway [69] in engineering L-serine-producing strains. However, these would incur growth arrest or cumbersome operation. In this work, the expression of *glyA* gene was dynamically controlled by the glycine-OFF riboswitch glyOFF6 in the serine-producing strain, resulting in an 104% increase of serine production with a yield of 85.40 mg g^−1^ glucose, and improved growth performance (a maximal OD_600_ of 2.55) (Fig. 5b). The expression of the *glyA* gene was dynamically controlled by the glycine-OFF riboswitch with the change of glycine level, realizing efficient resource utilization and an improvement of serine productivity, while also minimizing the need for chemical inducers. These results confirmed that it is possible to precisely regulate the expression of a key gene in an important metabolic pathway using our synthetic glycine-OFF riboswitch in *E. coli*.

Biosensors have been employed as high-throughput screening assays to identify directed evolution of single enzymes through coupling to the expression of fluorescent or selection markers for survival under selective conditions [70]. In this work, synthetic high-throughput screening platform based glyOFF6 is capable of enriching beneficial mutated plasmids under selective conditions due to the different growth rates of the high- and low-glycine producers. Strains with higher AGXT enzyme activity and glycine production were enriched under these selection conditions, while the neutral and negative mutations produced by error-prone PCR were removed by the screening platform. AGXT mutants, AGXT22 and AGXT26 with an increase of 58% and 73% enzyme activity were obtained through the synthetic high-throughput screening platform. AGXT26 included 14 mutations, some of which were positioned in PLP binding site or polypeptide binding sites. Polypeptide binding sites in AGXT are important for the stability of the protein [71–73]. We thus speculated that mutations Asp201Gly and Asp341Gly of AGXT26, as well as the Met38Thr, Lys51Arg and Arg241Ala of AGXT22 (Fig. 7d, Additional file 1: Fig. S11) were positioned in polypeptide binding sites, which might increase the stability of alanine-glyoxylate aminotransferase. Synonymous single-nucleotide polymorphisms (SNPs) do not produce altered amino acid sequences, and are not expected to change the function of the protein in which they occur [74]. However, it was confirmed that SNPs can subtly change the conformation and function of the protein [75, 76], and influence expression by affecting the degradation rate of the mRNA [77]. Therefore, four synonymous mutations (84th, 207th, 217th, 293rd) (Fig. 7d), especially 207th site with a higher-frequency codon (UCA) in AGXT26 might also contribute to the translation or protein conformation and thus improve the enzyme activity. The regulation of protein expression is complex, and enzyme activity is influenced by many factors [78]. It is possible that the combined effect of these mutations increases the enzyme activity of AGXT22 and AGXT26, more likely, some mutations are causal and the rest are hitchhikers, which need to further verify. Both enzyme activity and RNA levels might together determine that the increased production of 5-ALA in MG1655HAA22 and MG1655HAA26, which was significantly higher (22.58 and 25.30%) than in the control.

Glycine riboswitches exhibit single- or tandem-aptamer architectures, with distinct binding sites, followed by a single expression platform to regulate gene expression [48, 79]. To understand the relative contributions of each aptamer and its sequence toward gene regulation, top six synthetic glycine-ON riboswitches and top six synthetic glycine-OFF riboswitches (Additional file 1: Table S9) with the highest ON/OFF values, including glyON14 and glyOFF6, were sequenced and analyzed. Glycine binding sites (GBS) are the important role of affecting the binding of the sequence to glycine [40, 80]. Mutations U79C in glyOFF6, A56G in glyOFF5 as well as C143A, U177C, and U179A in glyOFF11 (Additional file 1: Table S10) were positioned in adjacent to the glycine binding sites, which might increase the amplitude values. The high frequency mutation U120A, A108G as well as A108U (Additional file 1: Table S10) might be helpful to alter the wild-type glycine-ON riboswitch into glycine-OFF riboswitch. Tandem glycine-OFF riboswitch variants were shown to exhibit preferential binding to the second aptamer [40], which can be explained the proportions of mutations occurring in the second aptamer of the six glycine-OFF riboswitches were higher than in the six glycine-ON riboswitches (Additional file 1: Fig. S4). Zhou et al., also demonstrated that mutation in the second aptamer would be more helpful for conformation alteration, and a synthetic glycine-OFF riboswitch was successfully obtained by introducing the G107U mutation into the second aptamer of a wild-type glycine-ON riboswitch [52].

Glycine riboswitches containing the P0/K-turn would affect glycine binding affinities and cooperation [29]. The mutations A4C in glyON4, A5G and A8U in glyON13, as well as C106U in glyON14 were positioned in the P0/K-turn, which might influence the glycine binding affinity and thus increase the detection range. Mutation located in the P1, P3A or P3B helices would influence the two aptamers to form a semisymmetric dimer through an extensive network of long-range tertiary interactions (the α, β, and γ interactions) [81, 82], A19G in GLYON14 and other mutation located (Additional file 1: Table S9) in these helices might also attribute to improve the performance of riboswitches. The detection ranges of glyON14 were wider than that of a previously reported in vivo* Clostridium* glycine riboswitch [52], which might be due to differences of genetic background, organism’s environment, expression plasmid and promoter. The in vivo sensitivity ranges partially matched those of in vitro experimental approaches or reports from other organisms [60]. Notably, non-rational error-prone PCR enables us to randomly introduce many mutations, but it can also introduce meaningless or detrimental changes when multiple mutations occur [83, 84]. The identified mutation sites in synthetic glycine-OFF and -ON riboswitches can form thousands of combinatorial mutants, and we did not further combine the mutations, nor did we verify the specific function of each mutation. The dynamic regulation displayed by each synthetic riboswitch, including both glycine-ON and -OFF riboswitches, was not the result of a single mutation, but possibly relied on multiple mutations working together or some specific function mutations. Although we did not verify each mutation point individually, these novel mutations may provide a valuable reference for the development of high-performance riboswitches or a valuable theoretical for other similar changes in riboswitch conformation. These synthetic riboswitches can be used as a gene expression regulation tool for metabolic engineering or synthetic biology.

## Conclusions

In summary, we successfully obtained a synthetic glycine-OFF riboswitch (glyOFF6) with significant repression in the presence of glycine, and an increased-detection-range glycine-ON riboswitch (glyON14) from a library based on the natural *B. subtilis* glycine-ON riboswitch. Synthetic glycine riboswitches were successfully used in tunable regulation, dynamic regulation and directed evolution of enzymes in *E. coli*. The novel mutations A56G, U79C, C143A, U177C, U179A U120A, A108G, and A108U in the glycine-OFF riboswitch, as well as A4C, A5G, A8U, A19G, C106U, and U179A in the glycine-OFF riboswitch, might have a positive effect on altering the wild-type conformation or improving the performance of the riboswitch. A high-throughput screening platform based on the synthetic glycine-OFF riboswitch was successfully used to screen AGXT26 with improved enzyme activity, which can be further applied to the synthesis 5-ALA and its derivatives. Overall, this work provides promising synthetic glycine-ON and -OFF riboswitches which have potential applications in directed evolution and dynamic metabolic regulation. This study presents an example of modifying a wild-type riboswitch from physiological significance to industrial significance, while also providing valuable information for further dissecting the functional domains of riboswitches and improving their performance.

## Materials and methods

### Bacterial strains, plasmids, media and regents

The primers, plasmids and *E. coli* strains used in this study are listed in Additional file 1: Tables S1, S2 and S3, respectively. Glycine riboswitch sequences used in this study are listed in Additional file 1: Tables S4. Information on the construction of plasmids and strains, reagents, as well as the composition of the medium, is available in the online supporting information.

### Construction and screening of glycine riboswitch and *agxT* gene library

The library of glycine riboswitch was constructed with Error-prone polymerase chain reaction (PCR) according to Instant Error-prone PCR Kit 2.0 (Additional file 1: Table S5). Glycine-ON and -OFF plasmid libraries were screened with the assay of ‘ON-SCREEN’ and ‘OFF-SCREEN’, respectively (Fig. 2). To improve the mutation frequency of the library, pUC18-BSTG was used as a template for the first round of Error-prone PCR, and then the purified PCR fragment was diluted to a concentration of 1 ng μL^−1^ for the other three rounds of Error-prone PCR. The template used in each round of Error-prone PCR was the last round of Error-prone PCR purification product. After four rounds of Error-prone PCR, the PCR products were assembled with the backbone fragment bslib-pUC18-BSTG containing the M1-37 promoter, dual selectable marker *tetA* and *gfp* gene. Subsequently, the glycine riboswitch library and backbone fragment were assembled together via Gibson assembly and transformed into *E. coli* MG1655 competent cell. All transformants from LB plate were harvested in M9 medium, half of strain was selected in the fresh M9 medium supplemented with 100 μg mL^−1^ ampicillin and 90 μM Ni^2+^ to remove glycine-OFF riboswitch, the clones were then cultured in the presence of 80 mM glycine to select glycine-ON riboswitch, following clones were cultured in fresh M9 medium supplemented with 100 μg mL^−1^ ampicillin, 80 mM glycine, and 15 μg mL^−1^ tetracycline to enrich glycine-ON riboswitches. Only clones displaying higher levels of *tetA* expression could survive the selection step, and the clones with the highest green fluorescent were sorted by FACS. Another half of strain was cultured in the presence of 100 μg mL^−1^ ampicillin, 90 μM Ni^2+^, and 80 mM glycine to remove glycine-ON riboswitches, the clones were then cultured in the fresh M9 medium supplemented with 100 μg mL^−1^ ampicillin but without glycine, following clones were cultured in M9 medium supplemented with 100 μg mL^−1^ ampicillin and 15 μg mL^−1^ tetracycline to enrich glycine-OFF riboswitches. Only clones displaying higher levels of *tetA* expression could survive the selection step, and the clones with the highest green fluorescent were then sorted by FACS (Details in supporting information). After three rounds of in vivo dual selection, cells with the highest fluorescence were sorted by FACS (fluorescence-activated cell sorting) from glycine-ON and -OFF riboswitches libraries. The sorted cells with the highest fluorescence were allowed to regenerate in SOC medium for 50 min and cultured on LB agar plates with or without 80 mM glycine and 90 μM NiCl_2_. The single colonies containing presumed glycine-ON or -OFF riboswitches were randomly selected and cultured in 96-deep-well plates containing 1 mL of M9 medium with or without 80 mM glycine, and the GFP/OD_600_ value of the was measured, respectively (for details see supporting information).

The *agxT* gene library was constructed according to the Controlled Error-prone PCR Kit instructions V1.4 (Additional file 1: Table s6). To improve the mutation frequency of the *agxT* gene library, the products of each round of Error-prone PCR were mixed and purified, and then used as a template for the next round of polymerase chain reaction. After four rounds of Error-prone PCR, the PCR products were assembled with the backbone fragment lib-pZY48-agxT containing the J23119 promoter, BBa_B0034 RBS by Gibson assembly and transformed into *E. coli* DH5α competent cells. The transformants were grown on LB agar plates. To reveal the expected mutations of *agxT* gene, 20 randomly picked clones were verified and sequenced. The single colonies on the agar plates were used for plasmid isolation to prepare the *agxT* plasmid libraries. The glyoxylate pathway was engineered to produce glycine by expressing *aceA* (encoding isocitrate lyase) and *agxT* (encoding alanine-glyoxylate aminotransferase, AGXT) from two plasmids (Fig. 6a, b). Plasmid libraries and pUC18-BSOFFTG were co-electroporated into fresh *E. coli* MG1655 cells. Then, the cells were diluted to an OD_600_ of 0.005 in 50 mL fresh M9 medium with 90 μM NiCl_2_. To prevent the adaptation of cells to NiCl_2_, cells were diluted in fresh selection medium when the OD_600_ value was over 0.5. After each enrichment cycle, these plasmids were extracted and electroporated again into fresh *E. coli* MG1655 cells. Cells from each enrichment cycle were also cultured on LB plates, identified by primers 48CK-F and 48CK-R, and sequenced to prove their mutation efficiency. And the third enrichment cycle cells were also cultured in 50 mL of M9 medium with 90 μM NiCl_2_ to measure the cellular growth rates. The single colonies were extracted from third enrichment cycle and sequenced, corresponding *agxT* mutant sequences were reconstructed into the pZY48. Beneficial AGXT mutants with higher activities in strains produced higher concentration of glycine, resulting in reporter gene *tetA* was inactivated, which would render the host cell insensitive to NiCl_2_. The reporter gene *tetA* was gradually suppressed with the increase of glycine produced by beneficial AGXT mutant. The strains containing the beneficial AGXT mutants grew much faster than the strains with low AGXT activities and gradually accumulated after three selection cycles (Fig. 6c) (for details see supporting information).

### Fluorescence measurement and enzyme activity assay

Fluorescence measurement was carried out according to a previous description (for details see supporting information section 2.8) [7]. The *K*_*d*_ value was estimated with OriginPro9.1 through non-linear regression of the Hill1 equation [39]. Alanine-glyoxylate aminotransferase activity was determined as described previously (as can be seen in supporting information section 2.9) [85]. The overnight culture of *E. coli* was diluted into fresh LB medium containing corresponding antibiotics and incubated at 37 °C until the optical density reached 0.6. Cells from 8 mL of fermentation broth were harvested by centrifugation at 13,800×*g* and 4 °C for 5 min. The cell pellet was resuspended in an equal volume of 50 mM potassium phosphate buffer (pH 7.0) and disrupted using an Ultrasonic Homogenizer (VC130PB, Sonics & Materials, Inc., Newtown, Conn.). The mixture was centrifuged at 13,800 × g and 4 °C for 10 min, and the supernatant was used for the enzyme activity assay. The transaminase activity was determined by following the formation of pyruvate from alanine with glyoxylate as the amino acceptor. Initial rate measurements were carried out in reaction mixtures comprising 10 mM alanine, 10 mM glyoxylate, 1 mM PLP (pyridoxal 5’-phosphate), 50 mM potassium phosphate (pH 7.0), and an appropriate amount of enzyme. The reactions were performed at 30 °C for 2–6 h and then quantified using HPLC. One unit of alanine-glyoxylate aminotransferase activity was defined as the amount of pyruvate (mmoL) produced per milliliter of enzyme solution extracted per OD_600_ cells for one minute.

### Fermentation

For 5-ALA shake flask fermentation, strains were incubated at 220 rpm and 37 °C in 500 mL flasks containing 50 mL of LB medium with corresponding antibiotics. Lactate fermentation was performed in 25 mL anaerobic bottles[86]. Serine fermentation was carried out according to a previous description [68].

### Analytical methods

The 5-ALA concentration was measured using modified Ehrlich’s reagent [87]. Glucose, lactate, acetate, formate, serine and pyruvate concentrations were determined by high performance liquid chromatography (HPLC, Agilent Technologies, USA) with RID (refractive index detector, Agilent Technologies, USA) [86]. Serine was quantified using automated precolumn derivatization with ortho-phthaldialdehyde (OPA), followed by HPLC with UV detection (Agilent Technologies, G1315D) [68].

## Supplementary Information

**Additional file 1:** Supporting Information Supporting Information of Materials and methods. **Table S1**. Primers used in this study. **Table S2**. Plasmids used in this study. **Table S3**. Strains used in this study. **Table S4**. Glycine riboswitches used in this study. **Table S5**. Instant Error-prone PCR Kit instructions 2.0. **Table S6**. Controlled Error-prone PCR Kit instructions V1.4. **Table S7**. *K*m values of mutation and wild-type glycine riboswitches. **Table S8**. Sequences of synthetic glycine-ON and -OFF riboswitches. **Table S9** Mutations and its locations of synthetic glycine-ON and -OFF riboswitches. Figure captions **Figure S1**. Glycine toxicity testing of E. coli MG1655 harboring glycine riboswitch in M9 medium with 0, 3, 7, 13, 27, 53, 80, 107, 426, 1705 mM glycine, respectively. **Figure S2**. Fluorescence intensity of strain MG1655-BS centrifugal pellets cultured in M9 medium supplemented with 0, 3, 7, 13, 27, 53, 80 mM glycine, respectively. **Figure S3**. Effect of tetA gene on glycine riboswitch and its function as genetic selection. **Figure S4**. Statistical analysis of mutation sites. **Figure S5**. Schematic diagram of long-range tertiary interactions (the α, β, γ, and δ interactions) in glycine riboswitch. **Figure S6**. Fermentation performances (a. OD_600_, b. Glucose, c. Formate, d. Acetate) of WB105-18A and WB105-18P in M9 medium supplemented with 0, 7, 13, 27, 80 mM glycine under anaerobic conditions with an initial OD_600_ of 0.05. **Figure S7**. Lactate maximum concentration of WB105-18A, WB105-15A and WB105-48A in M9 medium supplemented with 0, 1, 4, 7, 13, 27, and 80 mM glycine. **Figure S8**. D-Lactate production of W105-15A. **Figure S9**. Fermentation performances (a. Lactate, b. OD_600_, c. Glucose, d. Formate, e. Acetate) of WB105-15A and WB105-15P in M9 medium supplemented with 0, 7, 13, 27, 80 mM glycine under anaerobic conditions with an initial OD_600_ of 0.05. **Figure S10**. Fermentation performances (a. Lactate, b.OD_600_, c. Glucose, d. Formate, e. Acetate) of WB105-48A and WB105-48P in M9 medium supplemented with 0, 7, 13, 27, 80 mM glycine under anaerobic conditions with an initial OD<sub>600</sub> of 0.05. **Figure S11**. Statistical analysis of enrichment efficiency. **Figure S12**. Specific enzyme activities of 23 AGXT mutations, three AGXT library strains and control. **Figure S13**. Crystal structures of wild-type AGXT and mutant AGXT22. **Figure S14**. Distances between the site 20 in a subunit and the 43rd, 46th, 52nd, and 56th residue of another adjacent subunit on wild-type (left) and AGXT26 (right). **Figure S15**. Distance between the site 196 and the 7th residue of another adjacent subunit on wild-type (left) and AGXT26 (right).

## Data Availability

All data generated or analyzed during this study are included in this published article and its additional information files.

## Abbreviations

FACS: Fluorescence-activated cell sorting
AGXT: Alanine-glyoxylate aminotransferase
CRISPR: Clustered regularly interspaced short palindromic repeats
RBSs: Ribosome binding sites
FRET: Fluorescence resonance energy transfer
TFs: Transcription factors
Trp: Tryptophan
GBS: Glycine binding site
PLP: Pyridoxal 5-phosphate
SMHT: L-serine hydroxymethyltransferase
SNPs: Synonymous single-nucleotide polymorphisms
HPLC: High performance liquid chromatography
OPA: Ortho-phthaldialdehyde
RID: Refractive index detector
5-ALA: 5-Aminolevulinic acid
GFP: Green fluorescent protein
PCR: Polymerase chain reaction
GLU: Glucose
PGA: 3-Phosphoglycerate
PEP: Phosphoenolpyruvate
ISO: Isocitrate
AKG: α-Ketoglutaric acid
FUM: Fumaric acid
ICIT: Isocitrate
GOX: Glyoxylate
SUCCoA: Succinyl-CoA
SUC: Succinic acid
FUM: Fumaric acid
MAL: Malic acid
TCA cycle: Tricarboxylic acid cycle
PYR: Pyruvate
LAC: Lactate
Ac-CoA: Acetyl coenzyme A
Amp: Ampicillin
r: Resistance
glyON14: Glycine-ON riboswitch mutant
glyOFF6: Glycine-OFF riboswitch mutant
ori: Replicon
OAA: Oxaloacetic acid
PAP: 3-Phosphopyruvate
S3P: 3-Phosphoserine
MTPG: Methylene-tetrahydropteroyl polyglutamate
AMHDP: S-amino-methyldihydrolipoyl protein
GLY: Glycine
SER: Serine
PCR: Polymerase chain reaction
GCV: Glycine cleavage
