# Supplementary material for: Development and characterization of a glycine biosensor system for fine-tuned metabolic regulation in Escherichia coli
Source: Microb Cell Fact. 2022 Apr 7;21:56. doi: 10.1186/s12934-022-01779-4 (PMC8991567; doi:10.1186/s12934-022-01779-4)
Supplement: Supplementary file 1 — Additional file 1: Supporting Information Supporting Information of Materials and methods. Table S1. Primers used in this study. Table S2. Plasmids used in this study. Table S3. Strains used in this study. Table S4. Glycine riboswitches used in this study. Table S5. Instant Error-prone PCR Kit instructions 2.0. Table S6. Controlled Error-prone PCR Kit instructions V1.4. Table S7. Km values of mutation and wild-type glycine riboswitches. Table S8. Sequences of synthetic glycine-ON and -OFF riboswitches. Table S9 Mutations and its locations of synthetic glycine-ON and -OFF riboswitches. Figure captions Figure S1. Glycine toxicity testing of E. coli MG1655 harboring glycine riboswitch in M9 medium with 0, 3, 7, 13, 27, 53, 80, 107, 426, 1705 mM glycine, respectively. Figure S2. Fluorescence intensity of strain MG1655-BS centrifugal pellets cultured in M9 medium supplemented with 0, 3, 7, 13, 27, 53, 80 mM glycine, respectively. Figure S3. Effect of tetA gene on glycine riboswitch and its function as genetic selection. Figure S4. Statistical analysis of mutation sites. Figure S5. Schematic diagram of long-range tertiary interactions (the α, β, γ, and δ interactions) in glycine riboswitch. Figure S6. Fermentation performances (a. OD600, b. Glucose, c. Formate, d. Acetate) of WB105-18A and WB105-18P in M9 medium supplemented with 0, 7, 13, 27, 80 mM glycine under anaerobic conditions with an initial OD600 of 0.05. Figure S7. Lactate maximum concentration of WB105-18A, WB105-15A and WB105-48A in M9 medium supplemented with 0, 1, 4, 7, 13, 27, and 80 mM glycine. Figure S8. D-Lactate production of W105-15A. Figure S9. Fermentation performances (a. Lactate, b. OD600, c. Glucose, d. Formate, e. Acetate) of WB105-15A and WB105-15P in M9 medium supplemented with 0, 7, 13, 27, 80 mM glycine under anaerobic conditions with an initial OD600 of 0.05. Figure S10. Fermentation performances (a. Lactate, b.OD600, c. Glucose, d. Formate, e. Acetate) of WB105-48A and WB105-48P in M9 mediu [file 12934_2022_1779_MOESM1_ESM.docx]

**Development and Characterization of a Glycine Biosensor System for Fine-tuned Metabolic Regulation in *Escherichia coli***

Kun-Qiang Hong^1,2,3,4^, Jing Zhang^1,2,3,4^, Biao Jin^1,2,3,4^, Tao Chen^1,2,3,4^, Zhi-Wen Wang^1,2,3,4,*^

^1^ Department of Biochemical Engineering, School of Chemical Engineering and Technology, Tianjin University, Tianjin 300072, China;

^2^ Key Laboratory of Systems Bioengineering (Ministry of Education), Tianjin University, Tianjin 300072, China

^3^ SynBio Research Platform, Collaborative Innovation Center of Chemical Science and Engineering (Tianjin), Tianjin University, Tianjin 300072, China

^4^ Frontier Science Center for Synthetic Biology (Ministry of Education), Tianjin University, Tianjin 300072, China

*Corresponding author: Zhi-Wen Wang

Tel: +86-22-85356617; Fax: +86-22-85356617.

Address: Department of Biochemical Engineering, School of Chemical Engineering and Technology, Tianjin University, Tianjin 300072, People’s Republic of China. E-mail:zww@tju.edu.cn

E-mail: Kun-Qiang Hong: leo_hong92@163.com; Jing Zhang: zhangjing_2019@tju.edu.cn; Biao Jin: jinbiao@tju.edu.cn; Tao Chen: chentao@tju.edu.cn

**Supporting Information**

**Supporting Information of Materials and methods**

Table S1. Primers used in this study.

Table S2. Plasmids used in this study.

Table S3. Strains used in this study.

Table S4. Glycine riboswitches used in this study.

Table S5. Instant Error-prone PCR Kit instructions 2.0.

Table S6. Controlled Error-prone PCR Kit instructions V1.4.

Table S7. Km values of mutation and wild-type glycine riboswitches.

Table S8. Sequences of synthetic glycine-ON and -OFF riboswitches.

Table S9 Mutations and its locations of synthetic glycine-ON and -OFF riboswitches. **Figure captions**

Figure S1. Glycine toxicity testing of *E. coli* MG1655 harboring glycine riboswitch in M9 medium with 0, 3, 7, 13, 27, 53,80, 107, 426,1705 mM glycine, respectively.

Figure S2. Fluorescence intensity of strain MG1655-BS centrifugal pellets cultured in M9 medium supplemented with 0, 3, 7, 13, 27, 53, 80 mM glycine, respectively.

Figure S3. Effect of *tetA* gene on glycine riboswitch and its function as genetic selection.

Figure S4. Statistical analysis of mutation sites.

Figure S5. Schematic diagram of long-range tertiary interactions (the α, β, γ, and δ interactions) in glycine riboswitch.

Figure S6. Fermentation performances (a.OD_600_, b.Glucose, c. Formate, d.Acetate) of WB105-18A and WB105-18P in M9 medium supplemented with 0, 7, 13, 27, 80 mM glycine under anaerobic conditions with an initial OD_600_ of 0.05.

Figure S7. Lactate maximum concentration of WB105-18A, WB105-15A and WB105-48A in M9 medium supplemented with 0, 1, 4, 7, 13, 27, and 80 mM glycine.

Figure S8. D-Lactate production of W105-15A.

Figure S9. Fermentation performances (a.Lactate, b.OD_600_, c.Glucose, d.Formate, e.Acetate) of WB105-15A and WB105-15P in M9 medium supplemented with 0, 7, 13, 27, 80 mM glycine under anaerobic conditions with an initial OD_600_ of 0.05.

Figure S10. Fermentation performances (a.Lactate, b.OD_600_, c.Glucose, d.Formate, e.Acetate) of WB105-48A and WB105-48P in M9 medium supplemented with 0, 7, 13, 27, 80 mM glycine under anaerobic conditions with an initial OD_600_ of 0.05.

Figure S11. Statistical analysis of enrichment efficiency.

Figure S12. Specific enzyme activities of 23 AGXT mutations, three AGXT library strains and control.

Figure S13. Crystal structures of wild-type AGXT and mutant AGXT22.

Figure S14. Distances between the site 20 in a subunit and the 43rd, 46th,52nd, and 56th residue of another adjacent subunit on wild-type (left) and AGXT26 (right).

Figure S15. Distance between the site 196 and the 7th residue of another adjacent subunit on wild-type (left) and AGXT26(right).

**Materials and methods**

**Reagents**

DNA polymerase was purchased from Vazyme Biotech Co.,Ltd.(Beijing, China). GeneArt Gibson Assembly kit was purchased from New England Biolabs (Beijing, China). Oligonucleotide and gene were synthesized by Genewiz (Suzhou, China). Alanine, 5-aminolevulinic acid, glyoxylic acid, L-serine, D-lactate, acetate, formate, pyridoxal-5’ phosphate, PBS buffer, borate buffer, OPA (Phthaldialdehyde) derivative agent were purchased from Sigma-Aldrich (St. Louis, USA). Instant Error-prone PCR Kit was purchased from Beijing tianenze Biotechnology Co., Ltd. (Beijing, China). The FastDigest *Dpn*I was purchased from Thermo Fisher Scientific.

**Media**

Lysogrny Broth (LB) medium used for strain routinely grown processes contained (per liter) 10 g peptone, 5 g yeast extract, and 10 g NaCl. Agar plates were prepared with adding 2 %(*w/v*) agarose. Super Optimal broth with Catabolite repression (SOC) medium for strain recovery contained (per liter) 20 g peptone, 5 g yeast extract, 5 g NaCl, 1.86 g KCl, 9.5g MgCl_2_ and 4 g glucose. M9 minimal medium used in growth tests, and fermentation assays contained (per liter) 6.78 g Na_2_HPO_4_, 3 g KH_2_PO_4_, 0.5 g NaCl, 1.0 g NH_4_Cl, 0.24 g MgSO_4_, 0.011 g CaCl_2_, and 0.1 %(*v/v*) trace element solution[1]. The antibiotics ampicillin (100 μg mL^−1^), tetracycline (15 μg mL^−1^) and chloramphenicol (25 μg mL^−1^) were used when necessary.

**Plasmid construction**

Standard manipulation was carried out for polymerase chain reaction (PCR), DNA purification, enzyme digestion, and DNA ligation.

1. **Glycine riboswitch plasmids.** The high copy number plasmid pUC18 was used as the backbone for the construction of different glycine riboswitch plasmids. *gfp* gene (encoding green fluorescent protein) and plasmid backbone were amplified from plasmid pUC57-gfp and pUC18 using primers pUC18M1G-F containing the promoter M1-37 sequence and pUC18M1G-R, m1gpUC18-F/R, respectively. The purified PCR fragments were assembled using the GeneArt Gibson Assembly kit. Transformants were verified by colony PCR using primer pairs of pUC18-CKF/R, generating the control plasmid pUC18-M1G. Then, the fragments of *Bacillus subtilis* glycine riboswitch element BSN containing native promoter and its plasmid backbone bsnpUC18-GFP were amplified from the plasmid pUC57-BSN and pUC18-M1G with primers BSNG-F/R and bsnpUC18-F/R, respectively. Primers of pUC18-CKF/R were used to verify correct transformants, resulting in the glycine riboswitch plasmid pUC18-BSNGR. To construct another glycine riboswitch plasmid pUC18-BSGR, primers of BSG-F/R and bspUC18-F/R were used to amplify the *Bacillus subtilis* glycine riboswitch element BS without promoter and bspUC18-M1G. Other six glycine riboswitch plasmids, pUC18-FNNGR, pUC18-FNNGR, pUC18-SPNGR, pUC18-SPGR, pUC18-SPNGR, pUC18-SPGR were constructed in the similar way.
2. **pUC18-BSTG.** The dual selectable marker *tetA* (EMBL Accession: CAA23880.1) gene was amplified from the plasmid PUC-tetA with primers TET-F and TET-R. Primers pUC18BS-tetF and pUC18BS-tetR were used to amplify the fragment tetpUC18-BSGR from pUC18-BSGR. Then, two fragments were assembled together via Gibson assembly, resulting in pUC18-BSTG.
3. **pUC18-GlyON-ldhA, P15C-GlyON-ldhA and pZY48-GlyON-ldhA.** The *ldhA* gene (encoding lactate dehydrogenase) was amplified using the genomic DNA of *E. coli* W3101 as template with primers ldhA-F and ldhA-R. The PCR product was ligated into plasmid pUC18-GlyON14 with Gibson assembly, resulting in plasmid pUC18-BSGlyON-ldhA. The fused fragment of GlyON14 (mutanted *Bacillus subtilis* glycine-ON riboswitch) of pUC18-GlyON14 and *ldhA* gene were also inserted a low-copy number plasmids P15C **and pZY48**, resulting in plasmid P15C-BSGlyON-ldhA **and pZY48-GlyON-ldhA, respectively**.
4. **pZY48C-BSGlyOFF-glyA**. The *glyA* gene was amplified from the genomic DNA of *E. coli* MG1655 with primers glyA-F and glyA-R. The sequence of psc101 ori and chloramphenicol gene in pZY48 was amplified with primers CR48-F and CR48-R. With primers 18-glyAF and 18-glyAR, the plasmid skeleton with GlyOFF6 (another variant *Bacillus subtilis* glycine-OFF riboswitch) was amplified from PUC 18-GlyOFF 6. These three fragments were assembled together via Gibson assembly, resulting in pZY48C-BSGlyOFF-glyA.
5. **pUC18-HA.** The *Rhodococcus capsulatum hemA* gene (encoding 5-aminolevulinic acid synthase, NCBI version:O08374) was amplified from vector pXAC with primers RPHemA-F the and RPHemA-R. The plasmid backbone fragment pUC18-M137 with the M1-37 promoter was amplified from pUC18-M1G with corresponding primers 18-RPHemAF and 18-RPHemAR. The two fragments were assembled together to generate pUC18-rchemA. The gene *aceA* (encoding isocitrate lyase, NCBI version:NP_41843) under the M1-93 promoter control was assembled in pUC18, resulting in pUC18-aceA. Then, the fragments of M1-93 promoter and *aceA* gene were also inserted into the pUC18-rchemA, generating plasmid pUC18-HA.
6. **pZY48-****agxT.** The *Homo sapiens agxT* gene (encoding alanine-glyoxylate aminotransferase, NCBI version:P21549) under the control of J23119 promoter [2] and BBa_B0034 RBS (Ribosome Binding Site) [3], was optimized and synthesized in pUC18, resulting in pUC18- agxT. Then, the sequences of J23119 promoter, BBa_B0034 RBS and *agxT* gene were inserted into the pZY48 to obtain pZY48- agxT.

**Strain construction**

All strains used in this study were listed in Table S3. Different glycine riboswitch plasmids and pUC18-BSTG were transformed into the fresh *E. coli* MG1655, cultured on LB agar plate, resulting in corresponding strains. *ldhA* gene was deleted in *E. coli* WE269 according to previous method [4], generating strain *E. coli* W105. pUC18-BSGlyON-ldhA, P15C-BSGlyON-ldhA, pZY48-BSGlyON-ldhA and their corresponding control plasmids pUC18, P15C and pZY48 were transformed into the *E. coli* BW105, generating W105-18A, W105-15A, W105-48A, W105-18P, W105-15P an d W105-48P, respectively. pZY48C-BSGlyOFF-glyA and pZY48C were co-electroporated with pPk10 into fresh *E. coli* E4G2 cells, resulting in E4GS and E4GS, respectively. pUC18-rchemA and pZY48 were co-electroporated into *E. coli* MG1655 cells, resulting in the control strain MG1655K. pZY48-agxT, pZY48-agxT22, and pZY48-agxT26 were co-electroporated with pUC18-HAA into *E. coli* MG1655 cells, resulting in MG1655HAA, MG1655HAA22, and MG1655HAA26, respectively.

**Construction of glycine riboswitch library**

The *B. subtilis* glycine riboswitch library was constructed with primers BSgly-mutF and BSgly-mutR, which were designed according to the Instant Error-prone PCR Kit instructions 2.0 (details in supporting information Table S5). In each round of PCR, 4% of PCR products will still be wild-type, 84% of the PCR products will contain 1 to 5 mutation points, 12% of the PCR products will contain more over 6 mutation points. In order to improve the quality of the library, pUC 18-BSTG was used as a template for the first round of polymerase chain reaction, and then the purified fragment was diluted to a concentration of 1 ng μL^-1^ for the other three rounds of polymerase chain reaction. The template used in each round of PCR was the last round of PCR purification product. After four cycles of error-prone PCR, the PCR products were assembled with the backbone fragment bslib-pUC18-BSTG containing the M1-37 promoter, dual selectable marker *tetA* and *gfp* gene, respectively. bslib-pUC18-BSTG was amplified by 18BSGT-mutF and18BSgly-mutR from pUC18-BSTG. The linear PCR fragment was treated with FastDigest *Dpn*I (Thermo Fisher Scientific) at 37 °C for 15 min, inactivated at 80 °C for 5 min to remove the template plasmid pUC18-BSTG, and subsequently, the glycine riboswitch library and backbone fragment were assembled together via Gibson assembly and transformed into *E. coli* MG1655 competent cell. The transformants were grown on LB ager plates, and verified by primers pUC18-CKF and pUC18-CKR. Ten randomly selected clones were sequenced, revealing the expected random mutations at the targeted glycine riboswitch positions. All colonies on the agar plates were used for plasmid separation to prepare the glycine riboswitch plasmid library.

**Screening of glycine riboswitch library**

The screening workflow was shown in Figure 2. Glycine riboswitch library cells were washed from LB plates and diluted in 50 mL of fresh M9 medium and cultivated at 37 °C and 220 rpm for 12 h. These cells were used for the screening of glycine-ON and -OFF riboswitch.

**Screening of glycine-ON riboswitch.** The cells were diluted again to an OD_600_ of 0.005 in 50 mL of M9 medium supplemented with 90 μM NiCl_2_, and then cultured for 24 h for negative selection. Subsequently, the cells were washed and diluted to an OD_600_ of 0.005 in 50 mL fresh M9 medium containing 80 mM glycine and incubated for 12 h for the expression of *tetA* and *gfp*. The cells were diluted to an OD_600_ of 0.005 in 50 mL of M9 medium supplemented with 15 μg mL^-1^ tetracycline, and then cultured for 24 h for positive selection. The cells were washed again, and diluted to an OD_600_ of 0.005 in 50 mL fresh M9 medium with 80 mM glycine and incubated for 12 h, and subsequently, added with 15ug ml^-1^ tetracycline and cultured overnight. ~1.5×10^4^ cells with the highest green fluorescent were sorted by FACS (Fluorescence-activated cell sorting) from a total of ~2.5×10^7^ cells. The highest green fluorescent cells were recovered in SOC medium for 50 min and cultured on LB agar plates supplemented with 15 μg mL^-1^ tetracycline and 80 mM glycine. ~200 individual colonies were randomly selected and cultured in 96-deep-well plates with 1 mL of M9 medium supplemented with and without 80 mM glycine, and then measured the GFP/OD_600_ value of the plates with and without 80 mM glycine, respectively. The value measured in the 80 mM glycine-added plates was the glycine-ON riboswitch ‘ON’ state value, on the contrary, it was the ‘OFF’ state value. Strain MG1655-BS was the control. The colony with the highest ratio of ON/OFF in 96-deep-well plate was sequenced and rescreened in 50 mL of M9 medium with 0, 7, 13, 27, 80 mM glycine, respectively.

**Screening of glycine-OFF riboswitch.** The cells were also diluted to an OD_600_ of 0.005 in 50 mL of M9 medium supplemented with 90 μM NiCl_2_ and 80 mM glycine, and then cultured for 24 h for negative selection. Subsequently, the cells were washed and diluted to an OD_600_ of 0.005 in 50 mL fresh M9 medium without 80 mM glycine and incubated for 12 h for the expression of *tetA* and *gfp*. The cells were diluted to an OD_600_ of 0.005 in 50 mL of M9 medium supplemented with 15 μg mL^-1^ tetracycline and cultured for 24 h for positive selection. The cells were washed again and diluted to an OD_600_ of 0.005 in 50 mL fresh M9 medium without 80 mM glycine and incubated for 12h, and subsequently, added with 15 μg mL^-1^ tetracycline and cultured overnight. ~10^4^ cells with the highest green fluorescent were sorted by FACS from a total of ~10^7^ cells. The highest green fluorescent cells were recovered in SOC medium for 50 min and cultured on LB agar plates supplemented with 90 μM NiCl_2_ and 80 mM glycine. About 200 single colonies were randomly selected and cultured in 96-deep-well plates containing 1 mL of M9 medium supplemented with and without 80 mM glycine, and then the GFP/OD_600_ value of the plates with and without 80 mM glycine were measured, respectively. The value measured in the 80 mM glycine-added plates was the glycine-OFF riboswitch ‘OFF’ state value (OFF), on the contrary, it was the ‘ON’ state value (ON). The single colony with the highest ratio of ON/OFF in 96-deep-well plate was sequenced and rescreened in 50 mL of M9 medium with 0, 7, 13, 27, 80 mM glycine respectively.

**Construction and screening of *agxT* library**

The workflow of construction and screening was shown in Figure 6a*. Homo spices agxt* gene library was constructed with primers agxT-mutF and agxT-mutR, which were also designed according to the Controlled Error-prone PCR Kit instructions V1.4 (details in supporting information, Table s6). Different reactions were carried out to improve the mutation rate of the library. Different MnCl_2_ and dGTP dosage selection will introduce 1,3,5 and 7mutations into the sequence in each round of Error-prone PCR (Table s6). The products of each round of polymerase chain reaction were mixed and purified, and then used as a template for the next round of polymerase chain reaction. After four rounds of Error-prone PCR, the PCR products were assembled with the backbone fragment lib-pZY48-agxT containing the J23119 promoter, BBa_B0034 RBS, respectively. lib-pZY48-agxT was amplified by 48-agxtmutF and 48-agxtmutR from pZY48-agxT. The linear PCR fragment was also treated with FastDigest *Dpn*I (Thermo Fisher Scientific) at 37 °C for 15min, inactivated at 80 °C for 5 min to remove the template pZY48-agxT, and the *agxT* gene library and the skeleton fragment were assembled together by Gibson assembly and transformed into *E. coli* DH5α competent cells. The transformants were grown on LB agar plates. To reveal the expected mutations of *agxT* gene, 20 randomly selected clones were verified and sequenced. 20 selected clones all contained mutation sites. ～10^4^ colonies on the agar plates were used for plasmid isolation to prepare the *agxT* library. Plasmid libraries and pUC18-BSOFFTG were co-electroporated into fresh *E. coli* MG1655 cells. Then, the cells were diluted to an OD_600_ of 0.005 in 50 mL fresh M9 medium with 90 μM NiCl_2_. To prevent the adaptation of cells to NiCl_2_, cells were diluted in fresh selection medium when the OD_600_ value was over 0.5. After each enrichment cycle, these plasmids were extracted and electroporated again into fresh *E. coli* MG1655 cells. The strains containing the beneficial AGXT mutant with higher activity would produce higher concentration of glycine, resulting in reporter gene *tetA* was inactivated, which would render the host cell become insensitive to NiCl_2_. Therefore, the strains containing the beneficial AGXT mutant grew much faster than the strains with low AGXT activity. Beneficial mutations gradually accumulated after three selection cycles.

Cells from each enrichment cycle were also cultured on LB plates, identified by primers 48CK-F and 48CK-R, and sequenced to prove their mutation efficiency. And the third enrichment cycle cells were also cultured in 50 mL of M9 medium with 90 μM NiCl_2_ to measure the cellular growth rates. 23 colonies were extracted from third enrichment cycle and sequenced, corresponding *agxT* mutant sequences were reconstructed into the pZY48.

**Fluorescence measurement**

A single colony was transferred into 5 mL of liquid LB medium and grown overnight at 37 °C. Subsequently, a fresh culture in 50 mL M9 medium supplemented with various concentrations of glycine was inoculated to an initial OD_600_ of 0.01 and incubated at 37 °C and 220 rpm. Finally, the fluorescence of 500 μL of the diluted culture was monitored using a multimode microplate reader (483 nm excitation, 525 nm emission for GFP). The growth of cells was monitored by measuring the OD_600_ using a spectrophotometer (Beijing Puxi Universal Co Ltd, Beijing, China).

*agxT*

ATGGCTTCTCACAAACTGCTGGTTACCCCGCCGAAAGCTCTGCTGAAACCGCTGTCTATCCCGAACCAGCTGCTGCTGGGTCCGGGTCCGTCTAACCTGCCGCCGCGTATCATGGCTGCTGGTGGTCTGCAAATGATCGGTTCTATGTCTAAAGACATGTACCAGATCATGGACGAAATCAAAGAAGGTATCCAGTACGTTTTCC AGACCCGTAACCCGCTGACCCTGGTTATCTCTGGTTCTGGTCACTGCGCTCTGGAAGCTGCTCTGGTTAACGTTCTGGAACCGGGTGACTCTTTCCTGGTTGGTGCTAACGGTATCTGGGGTCAGCGTGCTGTTGACATCGGTGAACGTATCGGTGCTCGTGTTCACCCGATGACCAAAGACCCGGGTGGTCACTACACCCTGC AAGAAGTTGAAGAAGGTCTGGCTCAGCACAAACCGGTTCTGCTGTTCCTGACCCACGGTGAATCTTCTACCGGTGTTCTGCAACCACTGGACGGCTTCGGTGAACTGTGCCACCGTTACAAATGCCTGCTGCTGGTTGACTCTGTTGCTTCTCTGGGTGGTACCCCGCTGTACATGGACCGTCAGGGTATCGACATCCTGTACTCTGGTTCTCAGAAAGCTCTGAACGCTCCGCCGGGTACCTCTCTGATCTCTTTCTCTGACAAAGCTAAAAAAAAAATGTACTCTCGTAAAACCAAACCGTTCTCTTTCTACCTGGACATCAAATGGCTGGCTAACTTCTGGGGTTGCGACGACCAGCCGCGTATGTACCACCACACCATCCCGGTTATCTCTCTGTACTCTCTGCGTGAATCTCTGGCTCTGATCGCTGAACAGGGTCTGGAAAACTCTTGGCGTCAGCACCGTGAAGCTGCTGCTTACCTGCACGGTCGTCTGCAAGCTCTGGGTCTGCAACTGTTCGTTAAAGACCCGGCTCTGCGTCTGCCGACCGTTACCACCGTTGCTGTTCCGGCTGGTTATGACTGGCGTGACATCGTGAGCTACGTTATCGACCACTTCGACATCGAAATCATGGGTGGTCTGGGTCCGTCTACCGGTAAAGTTCTGCGTATCGGTCTGCTGGGTTGCAACGCTACCCGTGAAAACGTTGACCGTGTTACCGAAGCTCTGCGTGCTGCTCTGCAACACTGCCCGAAAAAAAAACTGTAA

*agxT26*

ATGGCTTCTCACAAACTGCTGGTTACCCCGCCGAAAGCTCTGCTGAAACCGCTGTCTACCCCGAACCAGCTGCTGCTGGGTCCGGGTCCGTCTAACCTGCCGCCGCGTATCATGGCTGCTGGTGGTCTGCAAATGATCGGTTCTATGTCTAAAGACATGTACCAGATCATGGACGAAATCAAAGAAGGTATCCAGTACGTTTTCCAGACCCGTAACCCGCTGACCCTGGTTATCTCTGGTTCTGGTCACTGTGCTCTGGAAGCTGCTCTGGTTAACGTTCTGGAACCGGGTGACTCTTTCCTGGTTGGTGCTAACGGTATCTGGGGTCAGCGTGCTGTTGACATCGGTGAACGTATCGGTGCTCGTGTTCGCCCGATGACCAAAGACCCGGGTGGTCACTACACCCTGCAAGAAGTTGAAGAAAGTCTGGCTCAGCACAAACCGGTTCTGCTGTTCCTGACCCACGGTGAATCTTCTACCGGTGTTCTGCAACCACTGGACGGCTTCGGTGAACTGTGCCACCGTTACAAATGCCTGCTGCTGGTTGACTCTGTTGCTTCTCTGGGTGGTACCCCGCTGTACATGTACCGTCAGGGTATCGGCATCCTGTACTCTGGTTCACAGAAAGCTCTGAACGCTCCGCCGGGTACTTCTCTGATCTCTTTCTCTGACAAAGCTAAAAAAAAAATGTGCTCTCGTAAAACCAAACCGTTCTCTTTCTACCTGGACATCAAATGGCTGGCTAACTTCTGGGGTTGCGACGACCTGCCGCGTATGTACCACCACACCATCCCGGTTATCTCTCTGTACTCTCTGCGTGAATCTCTGGCTCTGATCGCTGAACAGGGTCTGGAAAACTCTTGGCGTCAGCACCGTGAGGCTGCTGCTTACCTGCACGGTCGTCTGCAAGCTCTGGGTCTGCAACTGTTCGTTAAAGACCCGGCTCTGCGTCTGCCGACCGTTACCACCGTTGCTGTTCCGGCTGGTTATGACTGGCGTGACATCGTGAGCTACGTTATCGGCCACTTCGACATCGAAATCATGGGTGGTCTGGGTCCGTCTACCGGTAAAGTTCTGTGTATCGGTCTGCTGGGTTGCAACGCTACCCGTGAAAACGTTGACCGTGTTACCGAAGCTCTGCGTGCTGCTCTGCAACACTGCCCGAAAAAAGAACTGTAA

Table S1. Primers used in this study.

| Primer name | Sequence |
| --- | --- |
| pUC18M1G-F | GACAAGAGATAACAACGTTGATATAATTGAGCCACTGGCTCGTAATTTATTGTTTAAACCAGGAAACAGCTATGCGTAAAGGAGAAGAACTTTTCAC |
| pUC18M1G-R | CGGGGATCCTCTAGAGTCGACTTATTATTTGTATAGTTCATCCATGCCATG |
| m1gpUC18-F | ATGGCATGGATGAACTATACAAATAATAAGTCGACTCTAGAGGATCCCCGGG |
| m1gpUC18-R | CGAGCCAGTGGCTCAATTATATCAACGTTGTTATCTCTTGTCAACACCGCCAGAGATAAAAGCTTGGCACTGGCCGTCGTTTTAC |
| BSNG-F | GTAAAACGACGGCCAGTGCCAAGCTTCCCGCGAATCATATATTCTTTTAG |
| BSNG-R | GAAAAGTTCTTCTCCTTTACGCATCTAATTCCTCCCCTTTATCAACGGCGCAGCTAAAAAAC |
| bsnpUC18-F | GCGCCGTTGATAAAGGGGAGGAATTAGATGCGTAAAGGAGAAGAACTTTTCACTGGAG |
| bsnpUC18-R | CTAAAAGAATATATGATTCGCGGGAAGCTTGGCACTGGCCGTCGTTTTACAACGTC |
| BSG-F | GTTTAAACCAGGAAACAGCTTTGCTGTTGCCCAGCATATAG |
| BSG-R | GAAAAGTTCTTCTCCTTTACGCATCTAATTCCTCCCCTTTATCAAC |
| bspUC18-F | GTTGATAAAGGGGAGGAATTAGATGCGTAAAGGAGAAGAAC |
| bspUC18-R | CACTATATGCTGGGCAACAGCAAAGCTGTTTCCTGGTTTAAAC |
| FNNG-F | GTAAAACGACGGCCAGTGCCAAGCTTAAGAGTATAAAAAATTCCAGTTCCAAC |
| FNNG-R | GTGAAAAGTTCTTCTCCTTTACGCATTTTTTCTTATTGACAAAAATATAAAAAAG |
| fnnpUC18-F | CACTTTTTTATATTTTTGTCAATAAGAAAAAATGCGTAAAGGAGAAGAACTTTTCAC |
| fnnpUC18-R | CTGGAATTTTTTATACTCTTAAGCTTGGCACTGGCCGTCGTTTTAC |
| FNG-F | CGTAATTTATTGTTTAAACCAGGAAACAGCTTCTGAAATTAAAAAAG |
| FNG-R | AGTTCTTCTCCTTTACGCATTTTTTCTTATTGACAAAAATATA |
| fnpUC18-F | TATATTTTTGTCAATAAGAAAAAATGCGTAAAGGAGAAGAACTTTTC |
| fnpUC18-R | CTCTTGTCAACACCGCCAGAGATAATTTTTCTTATTGACAAAAATATAA |
| SPNG-F | GTTGTAAAACGACGGCCAGTGCCAAGCTTCAAGGATTGACAAATTTTTCATCAATTAAATAGGC |
| SPNG-R | CCAGTGAAAAGTTCTTCTCCTTTACGCATCGAAGACGCCAAAAGAAAAAAGC |
| spnpUC18-F | CTTTTTTCTTTTGGCGTCTTCGATGCGTAAAGGAGAAGAACTTTTCACTG |
| spnpUC18-R | GCCTATTTAATTGATGAAAAATTTGTCAATCCTTGAAGCTTGGCACTGGCCGTCGTTTTACAACG |
| SPG-F | GTTTAAACCAGGAAACAGCTCTTTTTTCTAGTTTAGGATCAG |
| SPG-R | GAAAAGTTCTTCTCCTTTACGCATCGAAGACGCCAAAAGAAAAAAGCTATC |
| sppUC18-F | GCTTTTTTCTTTTGGCGTCTTCGATGCGTAAAGGAGAAGAACTTTTCACTG |
| sppUC18-R | CCTAAACTAGAAAAAAGAGCTGTTTCCTGGTTTAAACAATAAATTAC |
| VCNG-F | GTTTATTTTTTAAGGGGAAATCACGATAACGACCCCCTTCTGCAATTTTTC |
| VCNG-R | GAAAAGTTCTTCTCCTTTACGCATGATTTCCCCTTAAAAAATAAACATAAG |
| vcnpUC18-F | GTTTATTTTTTAAGGGGAAATCATGCGTAAAGGAGAAGAACTTTTCACTG |
| vcnpUC18-R | GCAGAAGGGGGTCGTTATCGTGATTTCCCCTTAAAAAATAAAC |
| VCG-F | GTAATTTATTGTTTAAACCAGGAAACAGCTGCAAAAGACTTGCGCAACATTTCATC |
| VCG-R | GAAAAGTTCTTCTCCTTTACGCATGATTTCCCCTTAAAAAATAAACATAAG |
| vcpUC18-F | GTTTATTTTTTAAGGGGAAATCATGCGTAAAGGAGAAGAACTTTTCACTG |
| vcpUC18-R | CACCTACAAAAGAAATTAACTGATGAAATGTTGCGCAAGTCTTTTGCAGCTG |
| BS-MI64F | CAACGTTGATATAATTGAGCCCGTGTTTTCCCATGAAAAGTTTAAACCAGGAAACAGCTTTGCTGTTGCCCAGCATATAGTG |
| BS-MI64R | GGGCTCAATTATATCAACGTTGTTATCTCTTGTCAACACCGCCAGAGATAAAAGCTTGGCACTGGCCGTCGTTTTAC |
| BS-MI162F | GATAACAACGTTGATATAATTGAGCCTGTACAGTACTTCAATTTGTTTAAACCAGGAAACAGCTTTGCTGTTGCCCAGCATATAGTGATG |
| BS-MI162R | CAGGCTCAATTATATCAACGTTGTTATCTCTTGTCAACACCGCCAGAGATAAAAGCTTGGCACTGGCCGTCGTTTTAC |
| TET-F | CGTTGATAAAGGGGAGGAATTAGATGAATAGTTCGACAAAGATCGCATTG |
| TET-R | GTTCTTCTCCTTTACGCATAAGCTTAGAACCTCCTCCAGAACCTCCTCCAGATCCTCCTCCAGATCCACCTCCAAGCTAAGCACTTGTCTCCTGTTTACTCC |
| pUC18BS-tetF | GTAAACAGGAGACAAGTGCTTAGCTTGGAGGTGGATCTGGAGGAGGATCTGGAGGAGGTTCTGGAGGAGGTTCTAAGCTTATGCGTAAAGGAGAAGAACTTTTCACTG |
| pUC18BS-tetR | CGATCTTTGTCGAACTATTCATCTAATTCCTCCCCTTTATCAACGGC |
| BSgly-mutF | AGGAAACAGCTTTGCTGTTGCCCAGC |
| BSgly-mutR | CTAATTCCTCCCCTTTATCAACGGCGCAGC |
| 18BSGT-mutF | GCGCCGTTGATAAAGGGGAGGAATTAGATG |
| 18BSgly-mutR | GGGCAACAGCAAAGCTGTTTCCTGG |
| ldhA-F | GTTGATAAAGGGGAGGAATTAGATGAAACTCGCCGTTTATAGC |
| ldhA-R | GGGATCCTCTAGAGTCGACTTATTAAACCAGTTCGTTCGGGC |
| 18-ldhAF | CTGCCCGAACGAACTGGTTTAATAAGTCGACTCTAGAGGATCCCC |
| 18-ldhAR | GTGCTATAAACGGCGAGTTTCATCTAATTCCTCCCCTTTATCAACGGC |
| ldhA15-F | GATAACAATTTCACACAGAATTCTTATCTCTGGCGGTGTTGACAAG |
| ldhA15-R | GAGGATCCCCGGGTACCGAGCTCTTAAACCAGTTCGTTCGGGC |
| 15-ldhAF | CCTGCCCGAACGAACTGGTTTAAGAGCTCGGTACCCGGGGATC |
| 15-ldhAR | GTCAACACCGCCAGAGATAAGAATTCTGTGTGAAATTGTTATCCGC |
| Ck15-F | CACGTTACCCGGTTTACGCATAAGAATAAATAAATCCTGGTGTCCCTG |
| Ck15-R | CGTAGAAAAGATCAAAGGATCTTCACATCTCAATTGGTCTAGGTG |
| glyA-F | CGTTGATAAAGGGGAGGAATTAGATGTTAAAGCGTGAAATGAACATTG |
| glyA-R | GGGACACCAGGATTTATTTATTCTTATGCGTAAACCGGGTAACG |
| 18-glyAF | CACCTAGACCAATTGAGATGTGAAGATCCTTTGATCTTTTCTAC |
| 18-glyAR | GTTCATTTCACGCTTTAACATCTAATTCCTCCCCTTTATCAACGGC |
| RPHemA-F | GTTTAAACCAGGAAACAGCTATGAACTACGAAGCCTAC |
| RPHemA-R | CCCGGGGATCCTCTAGAGTCGACTTAGGCGGCTTTTGCGAGGCCCACGCGAGTCC |
| 18-RPHemAF | GTCGACTCTAGAGGATCCCCGGGTACCGAGCTCGAATTCGTAATCATGGTCATAG |
| 18-RPHemAR | AGCTGTTTCCTGGTTTAAACAATAAATTACGAGCCAGTGGCTCAATTATATC |
| pUC18-CKF: | CATTCGCCATTCAGGCTGCG |
| pUC18-CKR: | GCGGGCAGTGAGCGCAACGC |
| AceA-F | GTACTGAGAGTGCACCATATGC |
| AceA-R | CCCAACTTAATCGCCTTGC |
| 18H-AceAF | GGCGAAAGGGGGATGTGCT |
| 18H-AceAR | GCATCTGTGCGGTATTTCACAC |
| agxT-F | GTAACGCCAGGGTTTTCCCAGTCACTTACAGTTTTTTTTTCGGGCAGTG |
| agxt-R | CGAATACTAGAGAAAGAGGAGAAATACTAG |
| 48-agxtF | CTCCTCTTTCTCTAGTATTCGTGCTAGCATTATACCTAGGACTGAGCTAGCTGTCAAAAGCTTGGCTGTTTTGGCGG |
| 48-agxtR | GACTGGGAAAACCCTGGCGTTACCCAACGCATGCCTGCAGGTCGACTC |
| agxT-mutF | GTTGGGTAACGCCAGGGTTTTCCCAGTCAC |
| agxT-mutR | CGAATACTAGAGAAAGAGGAGAAATACTAG |
| 48-agxtmutF | CTCCTCTTTCTCTAGTATTCGTGCTAGCATTATACCTAGGACTGAGCTAGCTGTCAAAAGCTTGGCTGTTTTGGCGG |
| 48-agxtmutR | GACTGGGAAAACCCTGGCGTTACCCAACGCATGCCTGCAGGTCGACTC |
| 48CK-F | CAAGGCGCACTCCCGTTC |
| 48CK-R | GGCCCAGTCTTTCGACTGAG |

Table S2. Plasmids used in this study.

| Plasmid name | Genotype^a^ | Source |
| --- | --- | --- |
| pUC18 | *pColE1ori, Amp^r^* | laboratory stock |
| pUC57-BSNGR | *pColE1ori, Amp^r^, Wild-type Bacillus subtilis glycine riboswitch* | Genewiz synthsis |
| pUC57-SPNGR | *pColE1ori, Amp^r^, Wild-type Streptococcus pyogenes glycine riboswitch* | Genewiz synthsis |
| pUC57-VCNGR | *pColE1ori, Amp^r^, Wild-type Vibrio cholerae glycine riboswitch* | Genewiz synthsis |
| pUC57-FNNGR | *pColE1ori, Amp^r^, Wild-type Fusobacterium nucleatum glycine riboswitch* | Genewiz synthsis |
| pUC57-tetA | *pColE1ori, Amp^r^, tetA* | Genewiz synthsis |
| pUC57-gfp | *pColE1ori, Amp^r^, gfp* | Genewiz synthsis |
| pUC18-MIGFP | *pColE1ori, Amp^r^, gfp expression under M1-37 promoter* | this study |
| pUC18-BSNGR | *pColE1ori, Amp^r^, gfp expression under Wild-type Bacillus subtilis glycine riboswitch and itself native promoter* | this study |
| pUC18-BSGR | *pColE1ori, Amp^r^, gfp expression under Wild-type Bacillus subtilis glycine riboswitch and M1-37 promoter* | this study |
| pUC18-FNNGR | *pColE1ori, Amp^r^, gfp expression under Wild-type Fusobacterium nucleatum glycine riboswitch and itself native promoter* | this study |
| pUC18-FNGR | *pColE1ori, Amp^r^, gfp expression under Wild-type Fusobacterium nucleatum glycine riboswitch and M1-37 promoter* | this study |
| pUC18-VCNGR | *pColE1ori, Amp^r^, gfp expression under Wild-type Vibrio cholerae glycine riboswitch and itself native promoter* | this study |
| pUC18-VCGR | *pColE1ori, Amp^r^, gfp expression under Wild-type Vibrio cholerae glycine riboswitch and M1-37 promoter* | this study |
| pUC18-SPNGR | *pColE1ori, Amp^r^, gfp expression under Wild-type Streptococcus pyogenes glycine riboswitch and itself native promoter* | this study |
| pUC18-SPGR | *pColE1ori, Amp^r^, gfp expression under Wild-type Streptococcus pyogenes glycine riboswitch and M1-37 promoter* | this study |
| pUC18-BSTG | *pColE1ori, Amp^r^,tetA-gfp expression under Wild-type Bacillus subtilis glycine riboswitch and M1-37 promoter* | this study |
| pUC18-aceA | *pColE1ori, Amp^r^, aceA expression under M1-93 promoter* | this study |
| pUC18-BSTGOFF6-aceA | *pColE1ori, Amp^r^,tetA-gfp expression under Synthetic glycine-OFF riboswitch and M1-37 promoter, aceA expression under M1-93 promoter* | this study |
| pUC18-agxt | *pColE1ori, Amp^r^, agxT expression under J23119 promoter* | this study |
| pXAC | *Derived from pXMJ19, for overexpression  of hemA and hemC under the control of tac promote* | [5] |
| Puc18-rcHemA | *pColE1ori, Amp^r^, Rhodococcus capsulatum hemA expression under M1-37 promoter* | this study |
| pUC18-HA | *Puc18-rcHemA, aceA expression under M1-93 promoter* | this study |
| pUC18-GlyOFF6 | *Bacillus subtilis glycine-OFF riboswitch glyOFF6 with five mutation sites in pUC18-BSGR* | this study |
| pPK10 | *pBR322 replicon, Amp^r^, Ptrc- synRBS- pgk- synRBS- serA^Δ197^- synRBS- serB- synRBS- serC* | [6] |
| pZY48 | *Psc101 ori,Cm^r^* | laboratory stock |
| pZY48C-BSGlyOFF-glyA | *Psc101 ori,Cm^r^,Synthetic glycine-OFF glyOFF6 riboswitch fused with glyA* | this study |
| pZY48-agxT | *Psc101 ori,Cm^r^,agxT expression under J23119 promoter* | this study |
| pZY48-agxT 22 | *agxT with 7 mutation sites in pZY48-agxT* | this study |
| pZY48-agxT 26 | *agxT with 14 mutation sites in pZY48-agxT* | this study |
| pUC18-GlyON14 | *Bacillus subtilis glycine-ON riboswitch glyON14 with four mutation sites in pUC18-BSGR* | this study |
| P15C | *P15A replicon, Amp^r^* | laboratory stock |
| pUC18-BSGlyON-ldhA | *pColE1ori, Amp^r^,Synthetic glycine-ON riboswitch glyON14 fused with ldhA* | this study |
| P15C-BSGlyON-ldhA | *P15A replicon, Amp^r^,Synthetic glycine-ON riboswitch fused with ldhA* | this study |
| Pzy48-BSGlyON-ldhA | *Psc101 ori,Cm^r^, Synthetic glycine-ON riboswitch fused with ldhA* | this study |

^a^Amp: ampicillin, Cm: chloramphenicol, r: resistance.Table S3. Strains used in this study.

| *E. coli* name | Genotype | Source |
| --- | --- | --- |
| DH5α | *Strain K-12, F^-^ endA1 glnV44 thi-1 recA1 relA1 gyrA96 deoR nupG φ80dlacZΔM15 Δ(lacZYA-argF)U169 hsdR17(rk^-^, mk^+^)λ^-^* | laboratory stock |
| MG1655 | *Strain K-12, F^-^λ^-^rph-1* | laboratory stock |
| W3110 | *F-λ^-^ IN(rrnD-rrnE)1 rph-1* | laboratory stock |
| WE269 | *W3110 evolved strain* | [7] |
| WB105 | *WE269, ΔldhA* | this study |
| E4G2 | *MG1655, ΔsdaA, sdaB, ΔtdcG, Pgcv::PTrc-162, ΔglyA, Pkbl-tdh::Ptrc* | [6] |
| MG1655-pUC18 | *MG1655 Harboring pUC18* | this study |
| MG1655-pUC18-MIG | *MG1655 Harboring pUC18-MIG* | this study |
| MG1655- BSN | *MG1655 Harboring pUC18- BSNGR* | this study |
| MG1655-BS | *MG1655 Harboring pUC18- BSGR* | this study |
| MG1655- FNN | *MG1655 Harboring pUC18- FNNGR* | this study |
| MG1655-FN | *MG1655 Harboring pUC18- FNGR* | this study |
| MG1655-VCN | *MG1655 Harboring pUC18- VCNGR* | this study |
| MG1655-VC | *MG1655 Harboring pUC18- VCGR* | this study |
| MG1655-SPN | *MG1655 Harboring pUC18- SPNGR* | this study |
| MG1655-SP | *MG1655 Harboring pUC18- SPGR* | this study |
| MG1655-BSTG | *MG1655 Harboring pUC18- BSTG* | this study |
| MG1655-BS-ON14 | *MG1655 Harboring pUC18-GlyON14* | this study |
| MG1655-BS-OFF6 | *MG1655 Harboring pUC18-GlyOFF6* | this study |
| E4GD | *E4G2 Harboring pZY48c and ppk10* | this study |
| E4GS | *E4G2 Harboring pZY48c-glyoff-glyA and ppk10* | this study |
| MG1655-AA Lib1-3 (AGXT lib1-3) | *MG1655 Harboring pUC18-BSTGOFF6-aceA and pZY48-agxt-lib1(Third round enrichment)* | this study |
| MG1655-AA Lib2-2 (AGXT lib2-2) | *MG1655 Harboring pUC18-BSTGOFF6-aceA and pZY48-agxt-lib2 (Third round enrichment)* | this study |
| MG1655-AA Lib3-1(AGXT lib3-1) | *MG1655 Harboring pUC18-BSTGOFF6-aceA and pZY48-agxt-lib3 (Third round enrichment)* | this study |
| W105P | *WB105 Harboring pUC18* | this study |
| W105A | *WB105 Harboring pUC18-BSGlyON-IdhA* | this study |
| W105-15P | *WB105 Harboring P15C* | this study |
| W105-15A | *WB105 Harboring P15-BSGlyON-IdhA* | this study |
| W105-48P | *WB105 Harboring pZY48* | this study |
| W105-48A | *WB105 Harboring pZY48-BSGlyON-IdhA* | this study |
| MG1655K | *MG1655 Harboring pUC18-HA and pZY48* | this study |
| MG1655HAA | *MG1655 Harboring pUC18-HA and pZY48-agxT* | this study |
| MG1655HAA22 | *MG1655 Harboring pUC18-HA and pZY48-agxT22* | this study |
| MG1655HAA26 | *MG1655 Harboring pUC18-HA and pZY48-agxT26* | this study |

Table S4. Glycine riboswitches used in this study.

| Source | Glycine riboswitch sequence^a^ | Reference |
| --- | --- | --- |
| *Bacillus subtilis* | GTTTTCTGGATTTCTGCAGAGAGCTTGTACAGTTCCCAGTTTGCCCAAGGGCCATCGTTTTTCAGTCTTTCAGCAAATTCTTCAGGCCATTTTGCATCGTAGATCATTTCTGTATTCA**TTGCTT**TGAAACCGCCTT**TCTTATTCT**TCGATTCTTAAGATGAATAATATAAGAAAAATCTGCCCAAATTGCTGTTGCCCAGCATATAGTGATGATGGTAGGATATGAGTATGTATTTGATGTAAGATATTGCTATAGTATGTCCATAACAGCATGAAAATATGAGCGAATGACAGCAAGGGGAGAGACCTGACCGAAAACCTCGGGATACAGGCGCCGAAGGAGCAAACTGCGGAGTGAATCTCTCAGGCAAAAGAACTCTTGCTCGACGCAACTCTGGAGAGTGTTTGTGCGGATGCGCAAACCACCAAAGGGGACGTCTTTGCGTATGCAAAGTAAACTTTCAGGTGCCAGGACAGAGAACCTTCATTTTACATGAGGTGTTTCTCTGTCCTTTTTTGTATGTTTTTTAGCTGCGCCGTTGATAAAGGGGAGGAATTAG | [9] |
| *Streptococcus pyogenes* | AAAGTAAAATTAAAATAGCCCCCCAAAAAAGTTCATTAATACTAGCAATAAAATTTAACATAATAACACTCTCCTTTTCTATTTCAAATTTTACTGAATAAAAAAAGGTCTTTATTAAGACCTCTGAAATTAAAAAAGAATTATACAAATAAAAAATATATTTTAAAATATATTAAAAATATAAATTGCACAATTACTCCGTCCTTTTACCTGAGAGTTTAGGCTAAAATTAGCTTTGCTCCTTCGGTGTTATCTCTTAGATAAGCTCTCCAGAGGTTCGTCCAATATGAGTCCTTTTTACCTGAAAGATTTACTTCTTCGGTGTTTCATTAAAATGAAATCTCTCCTCATATCTTCATCCGATTATTTATTCAGTTTTTATGATGGAAATATTATCACTTTTTTATATTTTTGTCAATAAGAAAAA | [10] |
| *Vibrio cholerae* | ACGATAACGACCCCCTTCTGCAATTTTTCATTGGTACGGTAGCGAATCAACACAAACGCTA**ATGCTA**TCAGTAAGGCTATTGC**AATTAATCT**CACCATCTCCCGCCTCCAAGCGTGATTATCTTATAACACTTATACGTAGTTTAACGTCAGAGAAAAGTGGATTAGCAAAAGACTTGCGCAACATTTCATCAGTTAATTTCTTTTGTAGGTGACATCACATTTTCTTTTCGCTAGTATCCGCCTTGCAAATCGTTTTATTCAAGACGATTGTTCCGTTGAAGACTGCAGGAGAGTGGTTGTTAACCAGATTTTAACATCTGAGCCAAATAACCCGCCGAAGAAGTAAATCTTTCAGGTGCATTATTCTTAGCCATATATTGGCAACGAATAAGCGAGGACTGTAGTTGGAGGAACCTCTGGAGAGAACCGTTTAATCGGTCGCCGAAGGAGCAAGCTCTGCGCATATGCAGAGTGAAACTCTCAGGCAAAAGGACAGAGGAGTGAAAGGCCAATCTTTTAGTGAGCTCGCTAGAGCTCTGCTGTGCATTTTTCGCACCCTTTCTCTCTTCTCCTTATGTTTATTTTTTAAGGGGAAATC | [11] |
| *Fusobacterium nucleatum* | AAGAAAAATAGCAAAGAAGACCCCAAAAGTAAAGAGATTCCTTTTTTCTAGTTTAGGATCAGATATTTAAAAATAATAGCGATTTC**TTAAAG**CATTCCTTATTTTTT**TGCTATTTT**GGTTGACTTCATTCGTTTTTTGAGGTATGATAGTCCTAATTTAATAAACCGAATGATGTCATGCAGGAGAAGAATTTTTTTCGCCGAAGGAGTTATACTCTCAGGTGTTCAGTTTTTGAACGGGACTGTTTGATGGACGGACTTCTGGAGAGACCTTATTAGGCGCCGAAGGGGCAAGGCATACTGCTCAATCTCTCAGGCAAAAGGACAGAAGGTAAAATACAAACACCATTAAGAACAGTCTTAGTCTTTTTTGTGTTTGCTGTTTTATCATTGCTTCAGAAGTTGTCTCAAAGAAAGAGATAGCTTTTTTCTTTTGGCGTCTTCGATGACTTTT | [12] |

^a^The -35 and -10 consensus sequences were marked in orange, predicated by BPROM (Solovyev & Salamov 2011). Glycine riboswitches sequences were marked in blue.

Table S5. Instant Error-prone PCR Kit instructions 2.0^ab^

| Step | Name | Content |
| --- | --- | --- |
| 1 | Primers preparation | Primer length is designed to be 25-30 nt, Tm value is ~70 ℃, GC content is 45-60 %, and there are no hairpin structure. |
| 2 | Template preparation | Dilute the template to a concentration of 1 ng/μL |
| 3 | Standard PCR reaction system | 30 μL reaction system containing: 3 μL ERR-PCR Mix, 1 μL DNA template, 3 μL ERR-PCR dNTP, 3 μL ERR-PCR MnCl_2_, 1 μL each primer, 0.5 μL ERR-PCR Taq DNA Polymerase and 17.5 μL ddH_2_O. |
| 4 | Standard PCR reaction conditions | Pre-denaturation 94 ℃ 3 min, error-prone PCR reaction (94 ℃ 1 min, 45℃ 1 min, 72 ℃ 1 min), 50 cycles. |
| 5 | PCR products purification | DNA gel recovery kit^c^. |
| 6 | Increase mutation rate | Use the mutant purified DNA as a template for the next round of PCR reaction. |

^a^Suitable for mutations of DNA fragments below 1000 bp. In each round of PCR, 4% of PCR products will still be wild-type, 84% of the PCR products will contain 1 to 5 mutation points, 12% of the PCR products will contain more over 6 mutation points.

^b^https://www.tiandz.com.

^c^DNA gel recovery kit was purchased Shenggong Biological Engineering (Shanghai) Co., Ltd.

Table S6. Instant Error-prone PCR Kit instructions V1.4^ab^.

| Step | Name | Content | | | | |
| --- | --- | --- | --- | --- | --- | --- |
| 1 | Primers preparation | Primer length is designed to be 25-30 nt, Tm value is ~70 ℃, GC content is 45-60 %, and there are no hairpin structure. | | | | |
| 2 | Template preparation | Dilute the template to a concentration of 1ng/μL | | | | |
| 3 | MnCl_2_ and dGTP dosage selection | Expected number of mutations | 1 | 3 | 5 | 7 |
|  |  | MnCl_2_(μL) | 0 | 1 | 4 | 4 |
|  |  | dGTP (μL) | 0 | 0 | 1 | 3 |
| 4^c^ | Standard PCR reaction system^c^ | 30uL reaction system containing: 3 μL ERR-PCR Mix, 1 μL DNA template, *x* μL ERR-PCR dGTP, *y* μL ERR-PCR MnCl_2_, 1 μL each primer, 0.5 μL ERR-PCR Taq DNA Polymerase and supplement ddH_2_O to 30 μL. | | | | |
| 5 | Standard PCR reaction conditions | Pre-denaturation 94 ℃ 3 min, error-prone PCR reaction (94 ℃ 1 min, 45 ℃ 1min, 72 ℃ 1 min/1 kb) ,50 cycles. | | | | |
| 6 | PCR products purification | DNA gel recovery kit. | | | | |
| 7 | Increase mutation rate | Use the mutant purified DNA as a template for the next round of PCR reaction. | | | | |

^a^ Suitable for mutations of DNA fragments over 1000 bp.

^b^https://www.tiandz.com.

^c^The dosage of MnCl_2_ and dGTP is determined according to the number of expected mutations.

Table S7. *K_d_* values of mutant and wild-type glycine riboswitches

| Glycine riboswitch | *K_d_* (mM) | *P*** |
| --- | --- | --- |
| wild-type | 32 |  |
| glyON14 | 49 | 0.000 |

Table S8. Sequences of synthetic glycine-ON and -OFF riboswitches

| Mutation | Sequence^a^ |
| --- | --- |
| glyON3 | AAGTATTTGATGTAAGATATTGCTATAGTATGTCCATAACAGCATGAAAATATGAGCGAATGACAGCAAGGGGAGAGACCTGACCGAAAACCTCGGGATACAGG**T**GCCGAAGGAGCAAACTGCGGAGTGAATCTCTCAGGCAAAAGAACTCTTGCTCGACGCAACTCTGG**T**GAGTGTTTGTGCGGAT**A**CGCAAACCACCAAAGGGG**G**CGTCTTTGCGTATGCAAAGT**GG**ACTTTCAGGTGCCAGGACAGAGAACCTTCATTTTACATGAGGTGTTTCTCTGTCCTTTTTT |
| glyON4 | ATGTATTTGATGTAAGATATTGCTATAGTATGTCCATAACAGCATGAAAATATGAGCG**C**ATGACAGCAAGGGGAGAGACCTGACCGAAAACCTCGGGATACAGGCGCCGAAGGAGCAAACTGCGGAGTGAATCTCTCAGGCAAAAGAACTCTTGCTCGACGCAACTC**A**GGAGAGTGTTTGTGCGGATGCGC**T**AACCACCAAAGGGGACGTCTTTGCGTATGCAAAGTAAACTTTCAGGTGCCAGGACAGAGAACCTTCATTTTACATGAGGTGTTTCTCTGTCCTTTTTT |
| glyON9 | ATGTATTTGATGTAAGATATTGCTATAGTATGTCCATAACAGCATGAAAATATGAGCGAATGACAGCAAGGGGAGAGACCTGACCGAAAACCTCGGGATACAGGCGCCGAAGGAGCAAACTGCGGAGTGAATCTCTCAGGCAAAAGAACTCTAGCTCGACGCAACTCTGGAGAGTGTTTGTGC**A**GATGCGCAAACCACCAAAGGGGACGTCTTTGCGTATGCAAAGTAAAATTTCAGGTGCCAGGACAGAGAACCTTCATTTTACATGAGGTG**A**TTCTCTGTCCTTTTTT |
| glyON10 | ATGTATTTGATGTAAGATATTGCTATAGTATGTCCATAACAGCATGAAAATATGAGCGAATGACAGCAAGGGGAG**G**GACCTGACCGAAAACCTCGGGAT**T**CAGGCGCCGAAGGAGCAAACTGCGGAG**C**GAATCTCTCAGGCAAAAGAACTCTTGCTCGACGCAACTCTGGAGAGTGTTTGTGCGGATGCGCAAACCACCAAAGGGGACGTCT**A**TGCGTATGCAAAGTAAACTTTCAGGTGCCAGGACAGAGAACCTTCATTTTACATGAGGTGTTTCTCTATCCTTTTTT |
| glyON13 | ATGTATTTGATGTAAGATATTGCTATAGTATGTCCATAACAGCATGAAAATATGAGCGA**G**TG**T**CAGCAAGGGGAGAGACCTGACCGAAAACCTCGGGATACAGGCGCCGAAGGAG**T**AAACTGCGGAGTG**T**ATC**A**CTCAGGCAAAAGAACTCTTGCTCGACGCAACTCT**A**GGGAGTGTTTGTGCGGATGCGCAAACCACCAAAGGGGACGTCTTTGCGTATGCAAAGTAAACTT**A**CAGGTGCCAGGACAGAGAACCTTCATTTTACATGAGGTGTTTCTCTGTCCTTTTTT |
| glyON14 | ATGTATTTGATGTAAGATATTGCTATAGTATGTCCATAACAGCATGAAAATATGAGCGAATGACAGCAAGGGG**G**GAGACCTGACCGAAAACCTCGGGATAC**C**GGCGCCGAAGGAGCAAACTGCGGAG**A**GAATCTCTCAGGCAAAAGAACTCTTGCTCGAC**T**CAACTCTGGAGAGTGTTTGTGCGGATGCGCAAACCACCAAAGGGGACGTCTTTGCGTATGCAAAGTAAACTTTCAGGTGCCAGGACAGAGAACCTTCATTTTACATGAGGTGTTTCTCTGTCCTTTTTT |
| glyOFF5 | GCGAATGACAGCAA**T**GGGAGAGACCTGACCGAAAACCTCGGGATACAGGCGCCGA**G**GGAGCAA**T**CTGCGGAGTGAATCTCTCAGGCAAAAGAACTCTTGCTCGACGC**G**ACTCTGGAGAGTGT**C**TGTGCGGATGCGCA**T**ACCACCAAAGGGGACGTCTTTGCGTATGCAAAGTAAACTTTCAGGTGCCAGGACAGAGAACCTTCATTTTACATGAGGTGTTTCTCTGTCCTTTTTTGTA |
| glyOFF6 | ATGTATTTGATGTAAGATATTGCTATAGTATGTCCATAACAGCATGAAAATATGAGCGAATGACAGCAAGGGGAGAGACCTGACCGAAAACCTCGG**C**ATACAGGCGCCGAAGGAGC**T**AACTGCGGAGTGAATC**C**CTCAGGCAAAAG**G**ACTCTTGCTCGACGCAACTCTGGAGAG**A**GTTTGTGCGGATGCGCAAACCACCAAAGGGGACGTCTTTGCGTATGCAAAGTAAACTTTCAGGTGCCAGGACAGAGAACCTTCATTTTACATGAGGTGTTTCTCTGTCCTTTTTT |
| glyOFF8 | ATGTATTTGATGTAAGATATTGCTATAGTATGTCCATAACAGCATGAAAATATGAGCGAATGACAGCAAGGGGAGAGACCTGACCGAA**G**ACCTCGGGATACAGGCGCCGAAGGAGCAAACTGCGGAGTGAATCTCTCAGGCAAAAGAACTCTTGCTCGACGCAACTCTGGAGAG**A**GTTTGTGCGGATGCGCAAACCACCAAAGGGGACGTCTTTGC**A**TATGCAAAGTAAACTTTCAGGTGCCAGGACAGAGAACCTTCATTTTACATGAGGTGTTTCTCTGTCCTTTTTT |
| glyOFF11 | ATGTATTTGATGTAAGATATTGCTATAGTATGTCCATAACAGCATGAAAATATGAGCGAATGACAGC**G**AGGGGAGAGACCTGACCGA**T**AACCTCGGGA**A**ACAGGCGCCGAAGGAGCAAACTGCGGAGTGAATCTCTCAGGCAAAAGAACTCTTGCTCGACGCAACTCTGGAGAGTGTTTGTG**T**GGATGCGCAAACCA**A**CAAAGGGGACGTCTTTGCGTATGCAAAGTAAAC**C**T**A**CAGGTGCCAGGACAGAGAACCTTCATTTTACATGAGGTGTTTCTCTGTCCTTTTTT |
| glyOFF12 | ATGTATTTGATGTAAGATATTGCTATAGTATGTCCATAACAGCATGAAAATATGAGCGAATGACAGCAAGGGGAGAGAC**T**TGACCGAAAACCTCGGGATACAGGCGCCGAAGGAGCAAAC**A**GCGGAGTGAATCTCTCAGGCAA**G**AGAACTCTTGCTCGACGC**T**ACTCTGGAGAGTGTTTGTGCGGATGCGCAAACCACCAAAGGGGACGTCTTTGCGTATGCAAAGTAAACTTTCAGGAGCCAGGACAGAGAACCTTCATTTTACATGAGGTGTTTCTCTGTCCTTTTTT |
| glyOFF35 | ATGTATTTGATGTAAGATATTGCTATAGTATGTCCATAACAGCATGAAAATATGAGCGAATGACAGCAAGGGGAGAGACCTGACCGAA**G**ACCTCGGGATACAGGCGCCGAAGGAGCAAACTGCGGAGTGAATCTCTCAGGCAAAAGAACTCTTGCTCGACGCAACTCTGGAGAG**A**GTTTGTGCGGATGCGCAAACCACCAAAGGGGACGTCTTTGC**A**TATGCAAAGTAAACTTTCAGGTGCCAGGACAGAGAACCTTCATTTTACATGAGGTGTTTCTCTGTCCTTTTTT |

^a^The mutation positions were marked in redTable S9 Mutations and its locations of synthetic glycine-ON and -OFF riboswitches^a^

| Glycine-OFF riboswitch mutant | ON/OFF ratio^b^ | Mutant sites | Location | Glycine-ON riboswitch mutant | ON/OFF ratio^b^ | Mutant sites | Location |
| --- | --- | --- | --- | --- | --- | --- | --- |
| glyOFF5 | 4.6 | 15A-U | P1 | glyON3 | 4.3 | 50C-U | P2 |
|  |  | 56A-G | GBS |  |  | 106A-U | P1 |
|  |  | 64A-U | P3B |  |  | 133G-A | P2 |
|  |  | 108A-G | Linker seq |  |  | 152A-G | J2* |
|  |  | 123U-C | P2 |  |  | 173A-G | J2* |
|  |  | 138A-U | P2 |  |  | 174A-G | J2* |
| glyOFF6 | 5.5 | 42G-C | J1 | glyON4 | 4.5 | 4A-C | K-turn |
|  |  | 62A-U | J2 |  |  | 113U-A | P1 |
|  |  | 79U-C | GBS |  |  | 138A-U | P2 |
|  |  | 92A-G | P1 | glyON9 | 4.6 | 98U-A | P1 |
|  |  | 120U-A | P2 |  |  | 129G-A | J1* |
| glyOFF8 | 4.3 | 34A-G | J1* |  |  | 176C-A | P3a |
|  |  | 120U-A | P2 | glyON10 | 4.6 | 21A-G | JD |
|  |  | 162G-A | J2* |  |  | 45A-U | J1* |
| glyOFF11 | 4.2 | 13A-G | P1 |  |  | 73U-C | J2* |
|  |  | 33A-U | J1* |  |  | 158U-A | P3B |
|  |  | 44U-A | J1 | glyON13 | 4.9 | 5A-G | K-turn |
|  |  | 128C-U | P2 |  |  | 8A-U | P0/K-turn |
|  |  | 143C-A | GBS |  |  | 61C-U | J2* |
|  |  | 177U-C | GBS |  |  | 75A-U | J2* |
|  |  | 179U-A | GBS |  |  | 79U-A | P3a |
| glyOFF12 | 3.9 | 25C-U | P2 |  |  | 114U-A | P1 |
|  |  | 66U-A | P3b |  |  | 179U-A | GBS |
|  |  | 89A-G | JD | glyON14 | 5.9 | 19A-G | P1 |
|  |  | 108A-U | Linker seq |  |  | 47A-C | P2 |
| glyOFF35 | 3.9 | 34A-G | J1* |  |  | 73U-A | H2* |
|  |  | 120U-A | P2 |  |  | 106C-U | P0/K-turn |
|  |  | 162G-A | J2* |  |  |  |  |

^a^Base-pairing stems were labeled P0/kink-turn motif (P0/K-turn), P1, P2, and P3 with subsections labeled ‘a’ and ‘b’; and junction structures were labeled J1, J2 and JD with subsections labeled ‘*’; Linker seq, linker sequence; glycine binding sites were labeled as GBS, as also shown in Figure 3d,3e,3f.

^b^ON/OFF ratio was measured according to the experimental data from 96-deep-well plates.


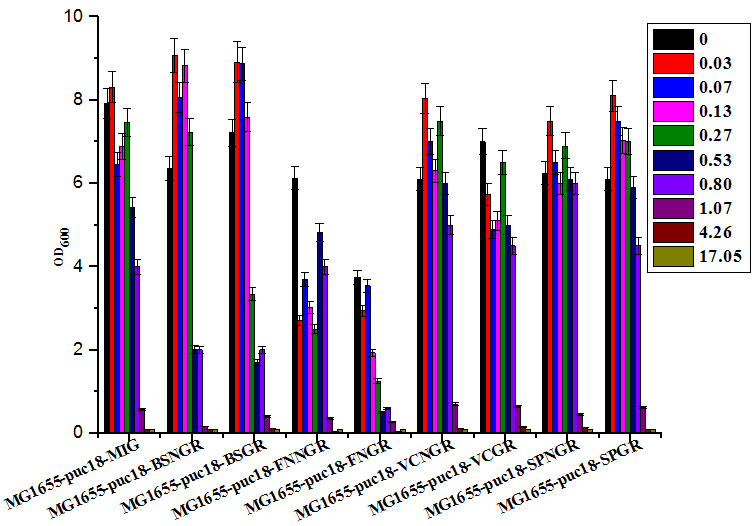


**Figure S1. Glycine toxicity testing of *E. coli* MG1655 harboring glycine riboswitch in M9 medium with 0, 3, 7, 13, 27, 53, 80, 107, 426, 1705 mM glycine, respectively.** All the data are the average values of three independent experiments.


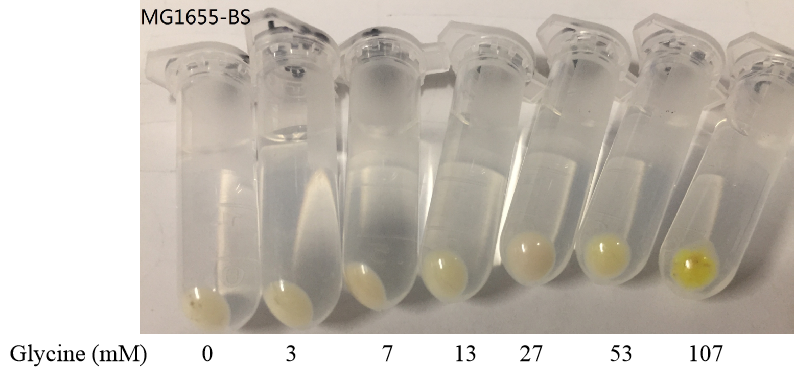


**Figure S2. Fluorescence intensity of strain MG1655-BSGR centrifugal pellets cultured in M9 medium supplemented with 0, 3, 7, 13, 27,53, 107 mM glycine, respectively.**


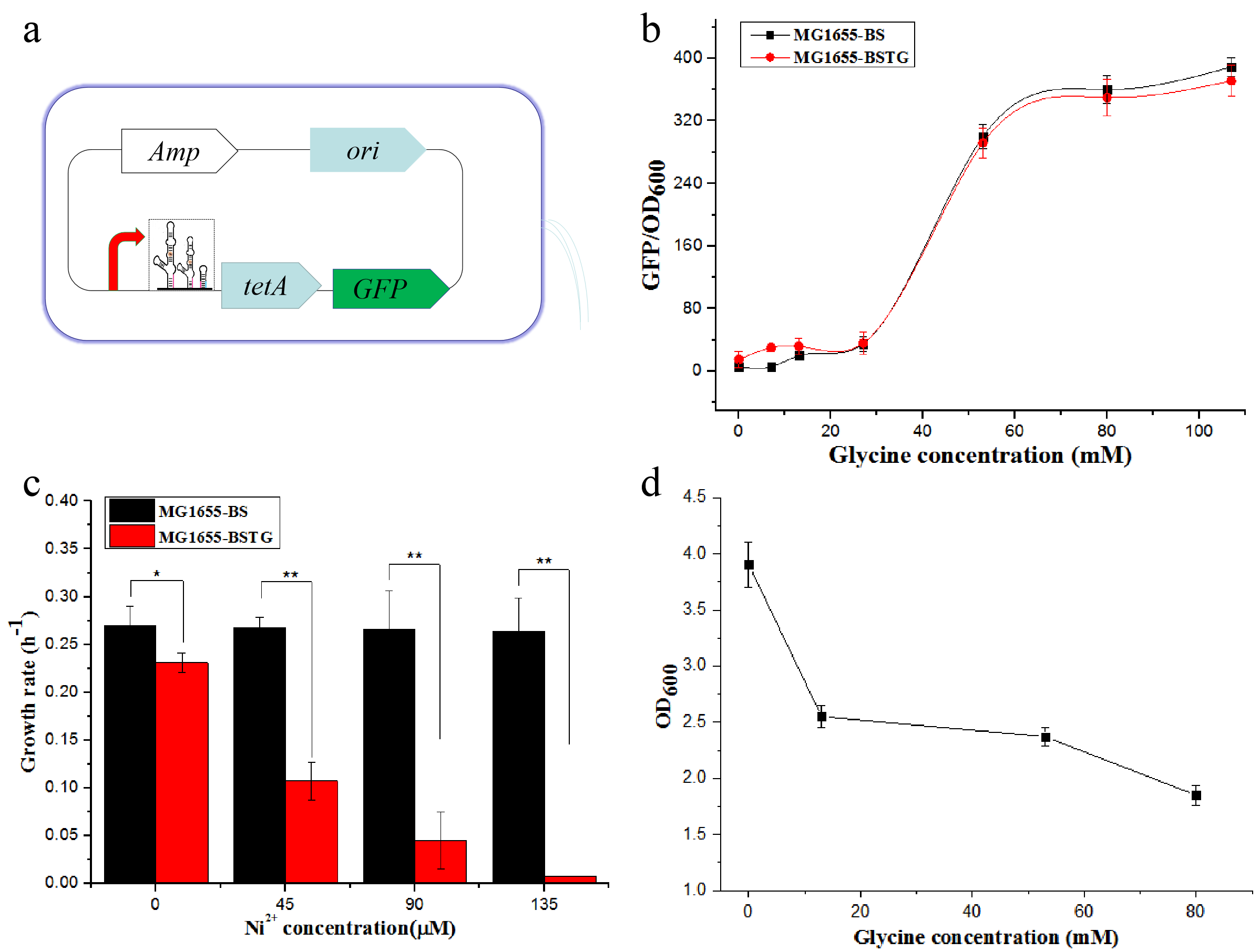


**Figure S3. Effect of *tetA* gene on glycine riboswitch and its function as genetic selection.** a. Diagram of pUC18-BSTG plasmid. *tetA* gene was fused with *gfp* under the control of *B. subtilis* glycine-ON riboswitch. The host cell would express green fluorescence by the expression of *gfp* and become resistant to tetracycline and sensitive to nickel by expression of *tetA* in the presence of glycine. The fluorescence intensity and the TetA were also accumulated with the increase of glycine. The nickel was pump-into cell by TetA, resulting in the growth of host cell be inhibited. b. Fluorescence intensity of strain MG1655-BS and MG1655-BSTG in M9 medium with 0,7, 13, 27, 53, 80, 107 mM glycine. c. Relative growth rate of strain MG1655-BS and MG1655-BSTG in M9 medium supplemented with 80 mM glycine and 0, 45, 90, 135 μM NiCl_2_. d. Maximum biomass of strain MG1655-BSTG in M9 medium supplemented with 90 μM NiCl_2_ and 0, 13, 53, 80 M glycine. All the data are the average values of three independent experiments. ***P<0.01; *P<0.05.*


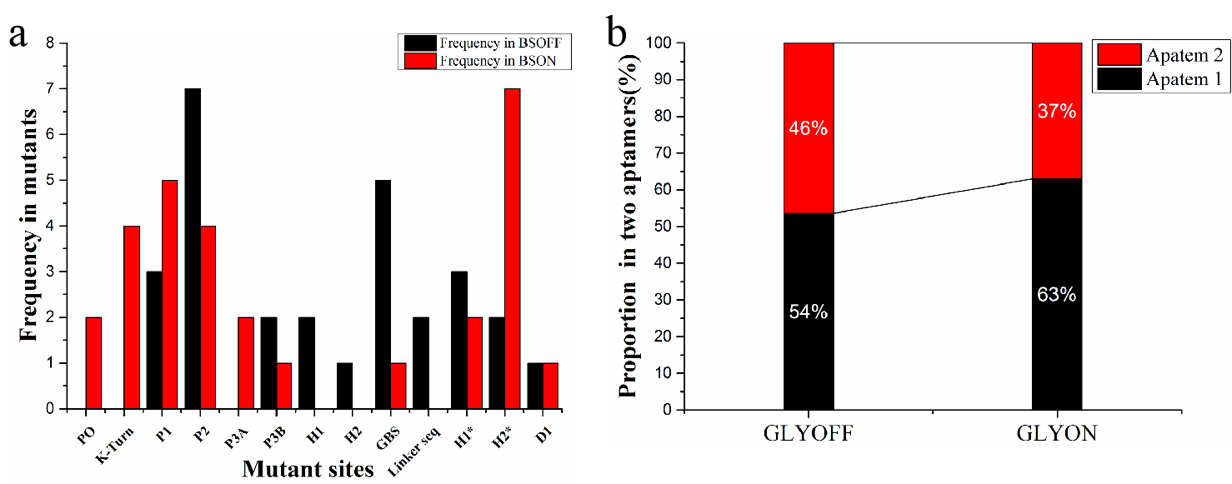


**Figure S4. Statistical analysis of mutation sites.** a. Frequency of mutation sites. b. Proportion of mutations frequency occurring in the two aptamers of the six Glycine-OFF riboswitches and six Glycine-ON riboswitches. The *y*-axis represents the percentage (%) of mutations frequency occurring in the two aptamers of the six Glycine-OFF riboswitches and six Glycine-ON riboswitches. The *x*-axis represents six Glycine-OFF riboswitches and six Glycine-ON riboswitches.


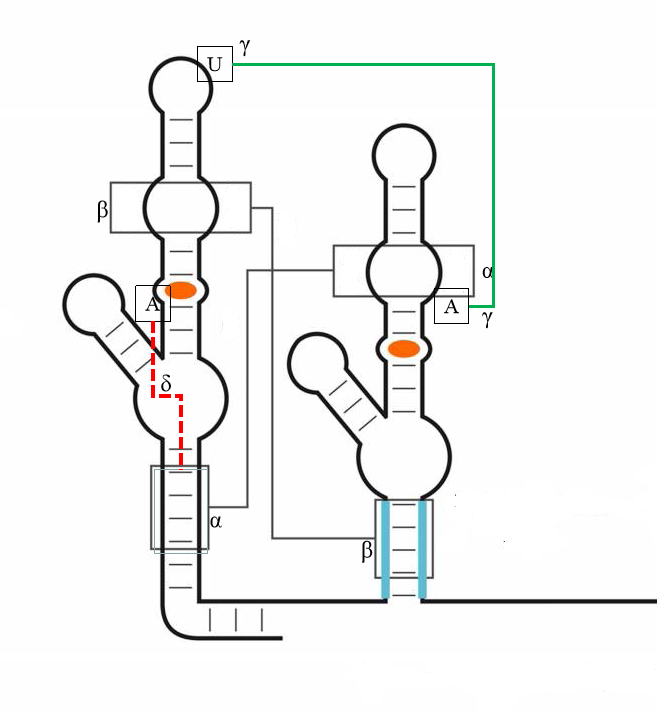


**Figure S5. Schematic diagram of long-range tertiary interactions (the α, β, γ, and δ interactions) in glycine riboswitch.**


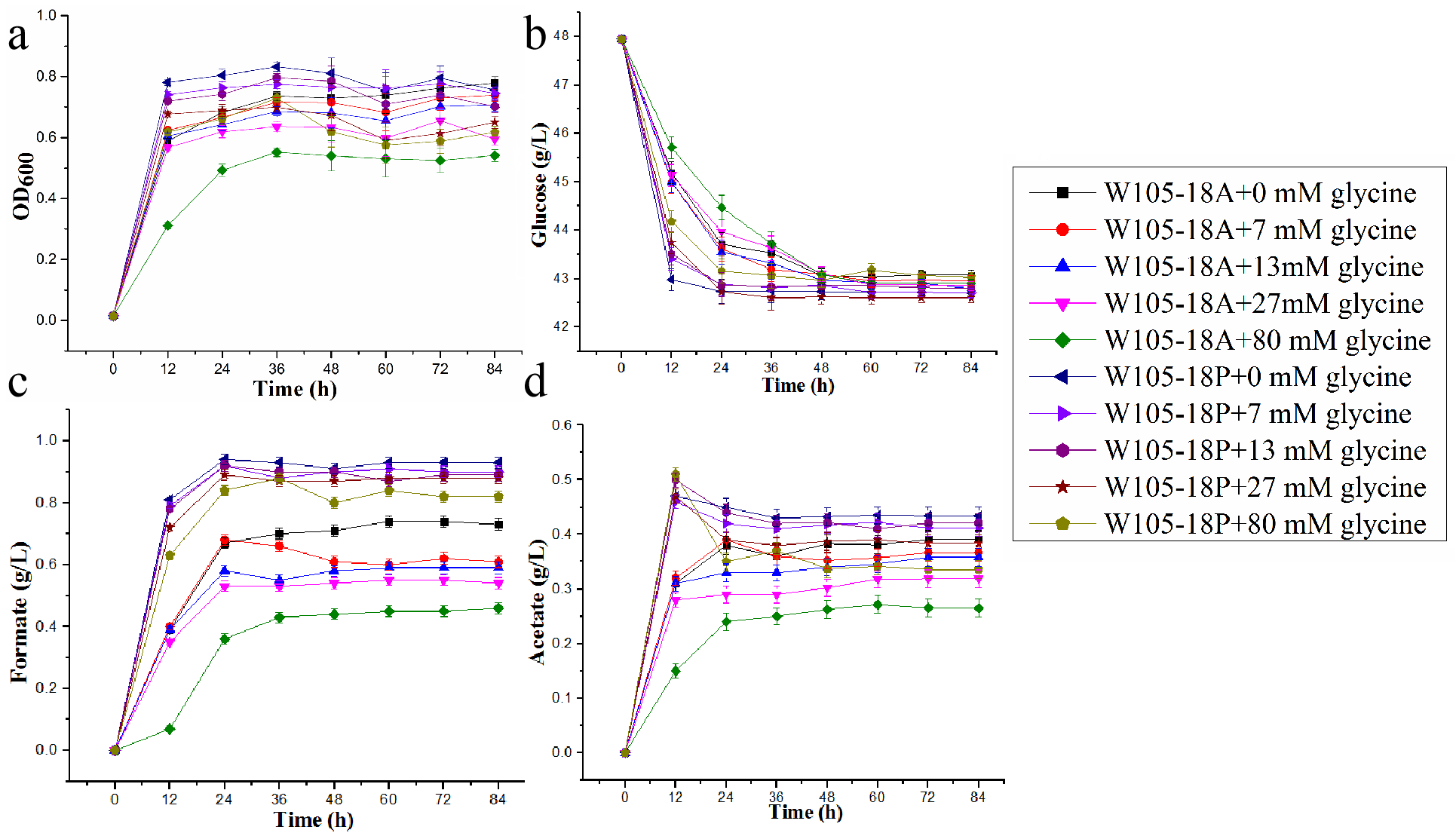


**Figure S6. Fermentation performances (a.OD_600_, b.Glucose, c. Formate, d.Acetate) of W105-18A and W105-18P** Error bars showed the standard deviations of triplicate samples.





**Figure S7. Lactate maximum concentration of W105-18A, W105-15A and W105-48A in M9 medium supplemented with 0, 1, 4, 7, 13, 27, and 80 mM glycine.** Error bars showed the standard deviations of triplicate samples.


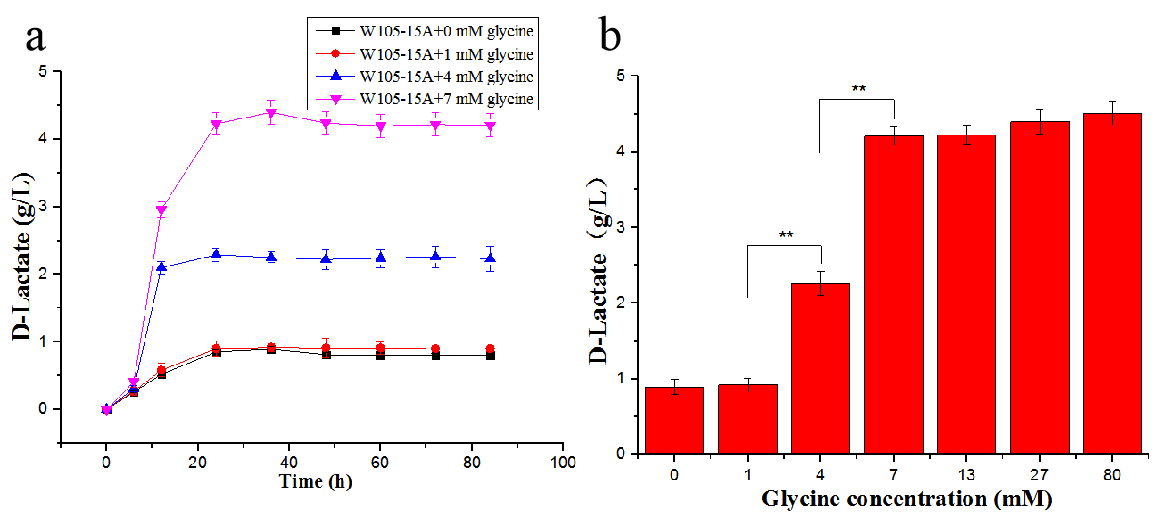


**Figure S8. D-Lactate production of W105-15A. a. D-Lactate production of W105-15A in M9 medium supplemented with 0, 1, 4, and 7 mM glycine b. D-Lactate maximum production of W105-15A in M9 medium supplemented with 0, 1, 4, 7, 13, 27, and 80 mM glycine.** Error bars showed the standard deviations of triplicate samples.***P<0.01.*

**
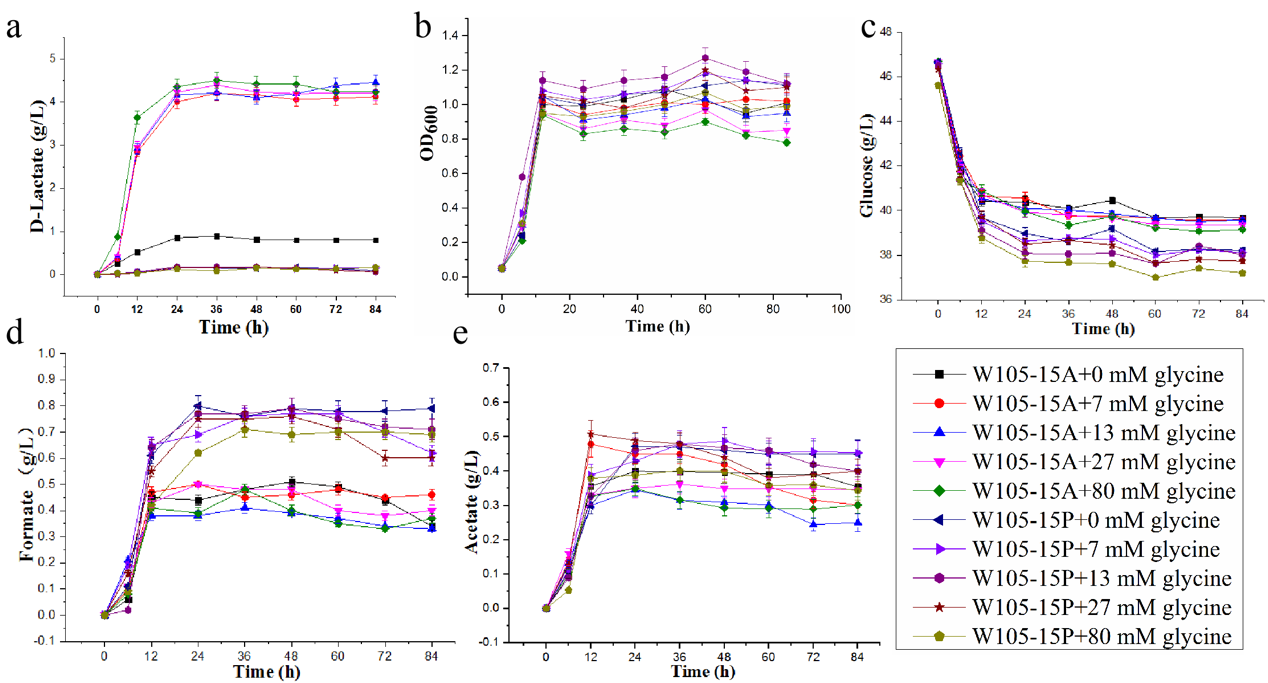
**

**Figure S9. Fermentation performances (a.OD_600_, b.Glucose, c. Formate, d.Acetate) of W105-15A and W105-15P in M9 medium supplemented with 0, 7, 13, 27, 80 mM glycine under anaerobic conditions with an initial OD_600_ of 0.05.** Error bars showed the standard deviations of triplicate samples.


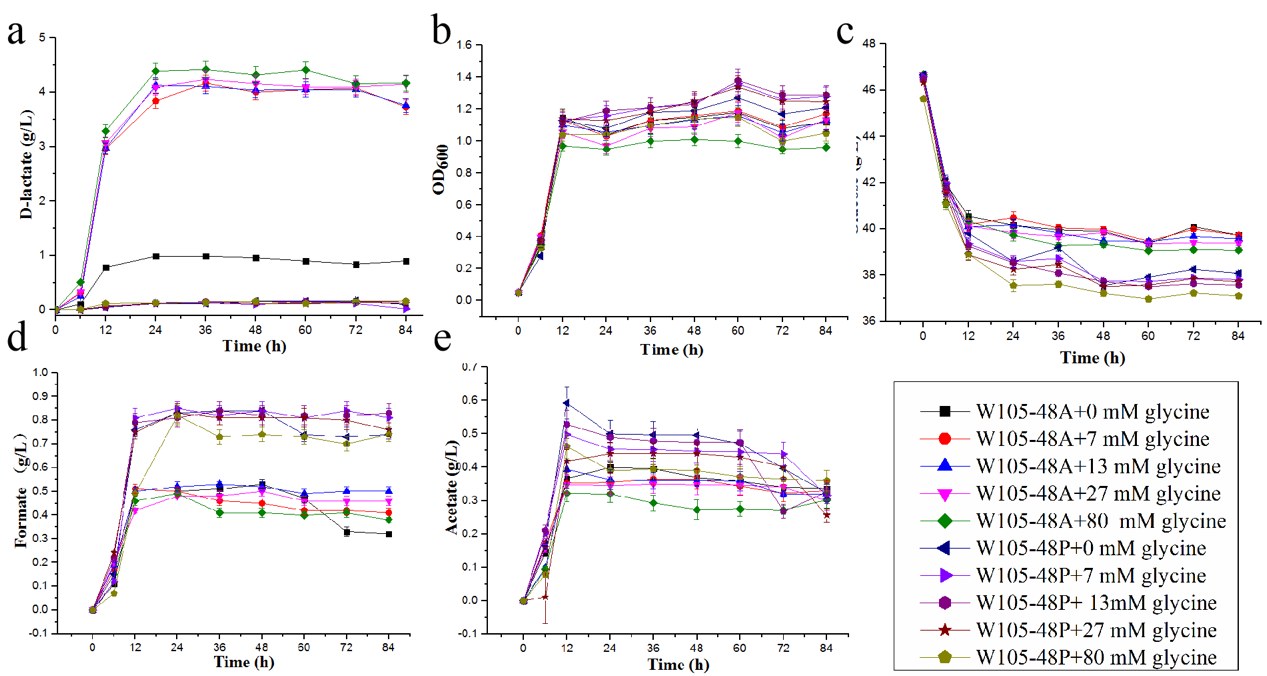


**Figure S10. Fermentation performances (a.OD_600_, b.Glucose, c. Formate, d.Acetate) of W105-48A and W105-48P in M9 medium supplemented with 0, 7, 13, 27, 80 mM glycine under anaerobic conditions with an initial OD_600_ of 0.05.** Error bars showed the standard deviations of triplicate samples.


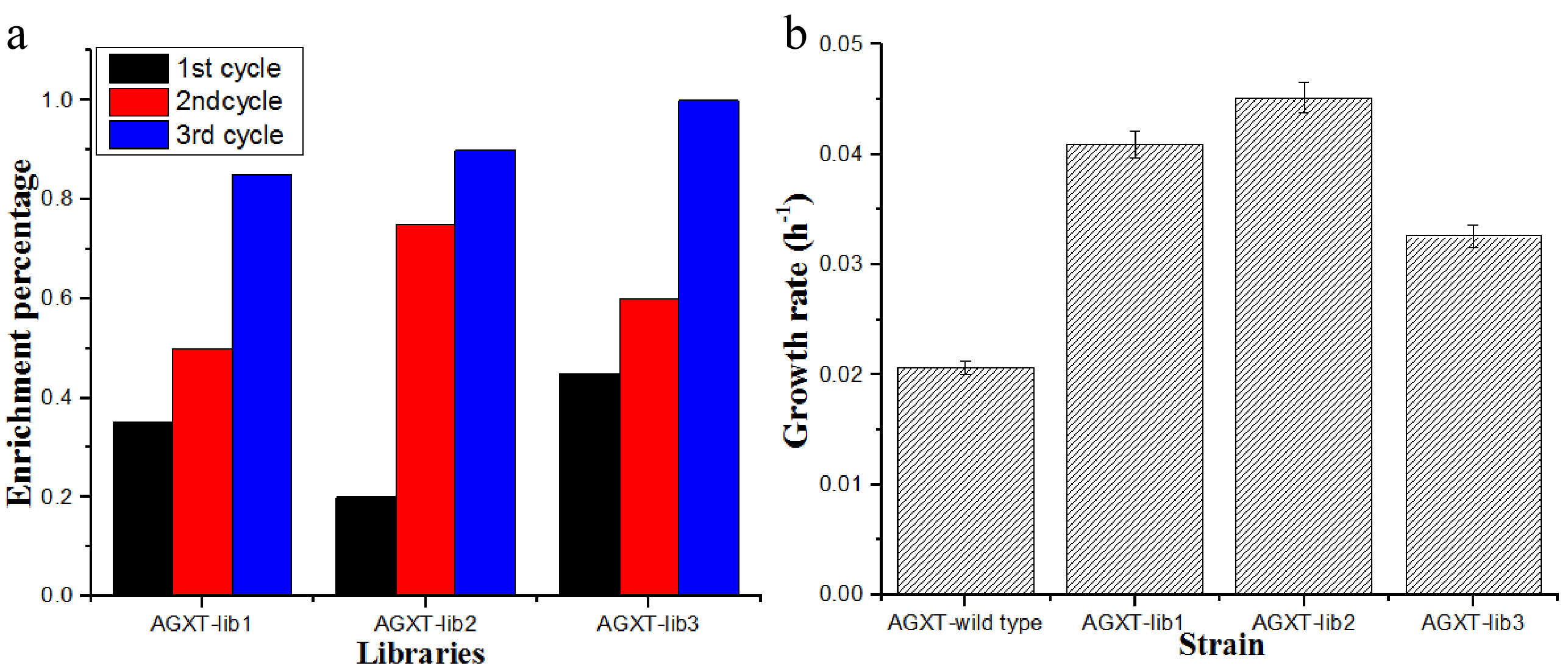


**Figure S11. Properties of the enriched cultures.** a Population analysis of each cycle. The *y*-axis represents the percentage of each variant in the total population. The *x*-axis represents the enrichment cycle, where each enrichment cycle corresponds to three AGXT libraries. b Growth rate of the strains in the third round of the last cycle.



**Figure S12. Specific enzyme activities of 23 AGXT mutations, three AGXT library strains and control.**
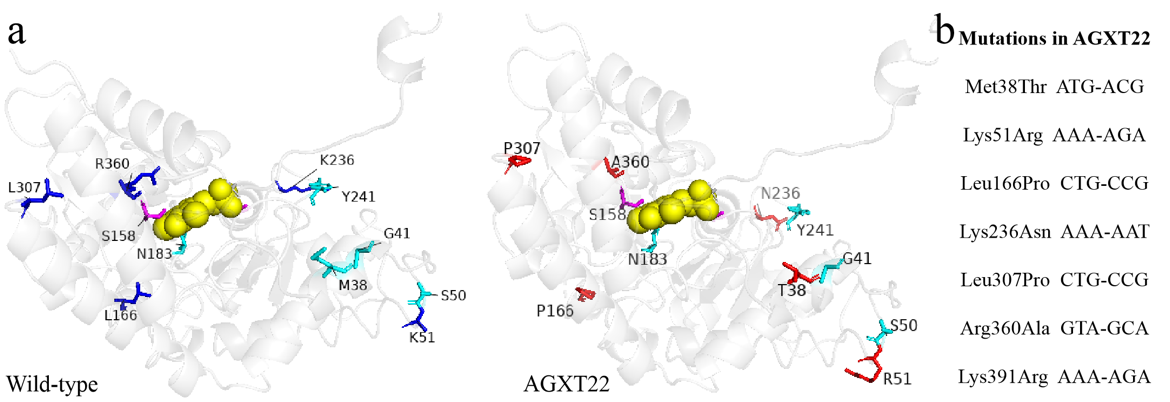


**Figure S13. Crystal structures of wild-type AGXT and mutant AGXT22.** (a) Six wild type sites were marked on the left, mutation sites in AGXT22 were marked on the right. (b) Details of the mutant sites in AGXT22. Crystal structure was showed as gray cartoon, wild type sites were showed as blue stick, mutant sites were showed as red stick, chemical binding sites residues were showed as magenta stick, polypeptide binding sites residues were showed as cyan sticks, LPL (Pyridoxal Phosphate) was showed in yellow balls. The annotation was carried out with PyMOL software.
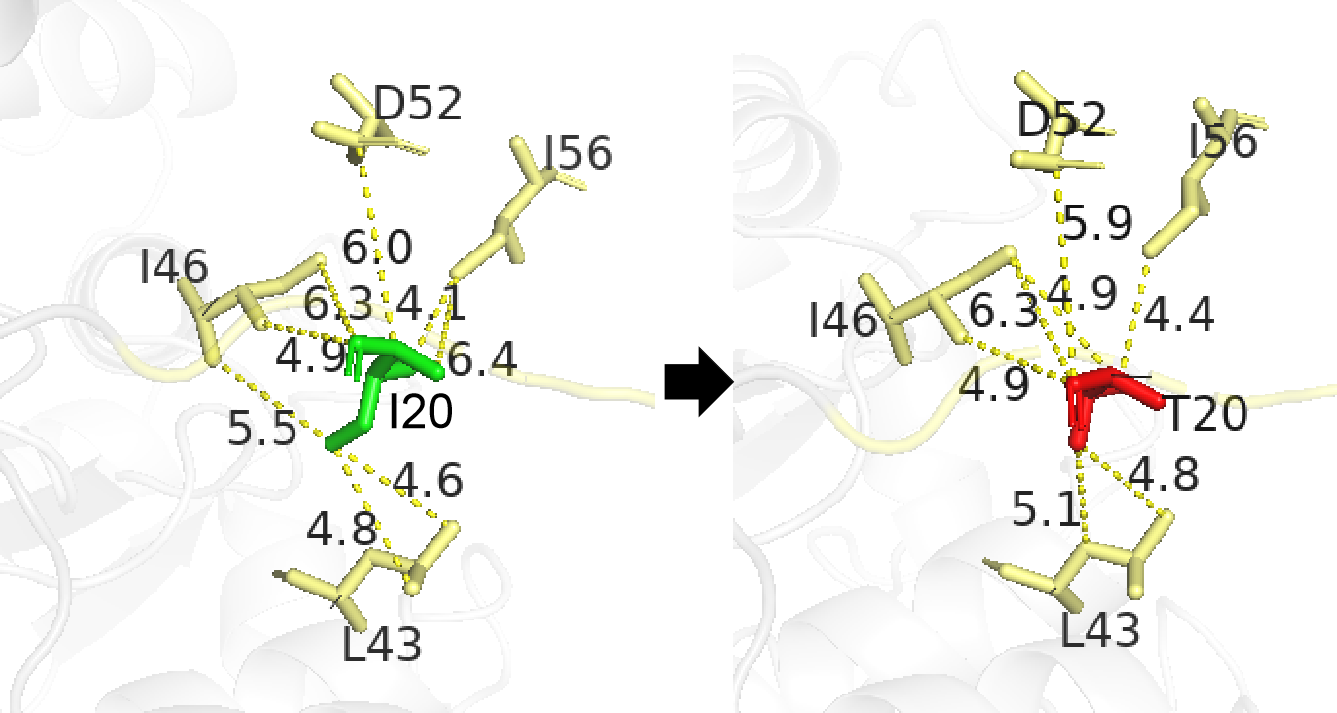


**Figure S14. Distances between the site 20^th^ in a subunit and the 43^rd^, 46^th^, 52^nd^, and 56^th^ residue of another adjacent subunit on wild-type (left) and AGXT26 (right).** I20 in wild type AGXT was showed as green stick, and mutation site T20 in AGXT26 was showed as red stick, 43^rd^, 46^th^, 52^nd^, and 56^th^ residues were showed as paleyellow. The annotation in crystal structure was carried out with PyMOL software.
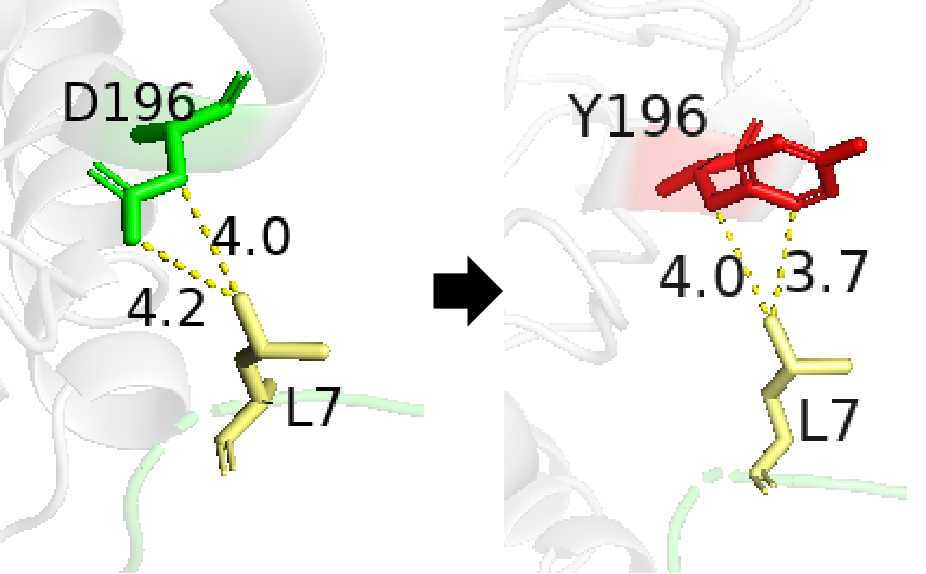


**Figure S15. Distance between the site 196 and the 7^th^ residue of another adjacent subunit on wild-type (left) and AGXT26(right).** D196 in wild type AGXT was showed as green stick, and mutation site Y196 in AGXT26 was showed as red stick, L7 was showed as paleyellow. The annotation in crystal structure was carried out with PyMOL software.References

1 Zhang X, Jantama K, Moore J C, Jarboe L R, Shanmugam K T, Ingram L O, Metabolic evolution of energy-conserving pathways for succinate production in *Escherichia coli*. Proc Natl Acad Sci U S A. 2009;106(48):20180-5.

2 Liu R M, Liang L L, Freed E, Chang H, Oh E, Liu Z Y, Garst A, Eckert C A, Gill R T, Synthetic chimeric nucleases function for efficient genome editing. Nat Commun. 2019;10(5524):1-10.

3 Bryksin A V, Bachman H N, Cooper S W, Balavijayan T, Blackstone R M, Du H, Jenkins J P, Haynes C L, Siemer J L, Fiore V F, Barker T H, One primer to rule them all: universal primer that adds BBa_B0034 ribosomal binding site to any coding standard 10 BioBrick. ACS Synth Biol. 2014;3(12):956-9.

4 Kuhlman T E, Cox E C, Site-specific chromosomal integration of large synthetic constructs. Nucleic Acids Res. 2010;38(6):e92.

5 Feng L L, Zhang Y, Fu J, Mao Y F, Chen T, Zhao X M, Wang Z W, Metabolic engineering of *Corynebacterium glutamicum* for efficient production of 5-aminolevulinic acid. Biotechnol Bioeng. 2016;113(6):1284-93.

6 Zhang Y, Kang P, Liu S, Zhao Y J, Wang Z W, Chen T, *glyA* gene knock-out in *Escherichia coli* enhances L-serine production without glycine addition. Biotechnol Bioproc E. 2017;22(4):390-6.

7 Wang B W, Zhang X X, Yu X L, Cui Z Z, Wang Z W, Chen T, Zhao X M, Evolutionary engineering of *Escherichia coli* for improved anaerobic growth in minimal medium accelerated lactate production. Appl Microbiol Biot. 2019;103(5):2155-70.

8 Lu J, Tang J L, Liu Y, Zhu X N, Zhang T C, Zhang X L, Combinatorial modulation of *galP* and *glk* gene expression for improved alternative glucose utilization. Appl Microbiol Biot. 2012;93(6):2455-62.

9 Babina A M, Lea N E, Meyer M M, In vivo behavior of the tandem glycine riboswitch in *Bacillus subtilis*. mBio. 2017;8(5):1-16.

10 Khani A, Popp N, Kreikemeyer B, Patenge N, A glycine riboswitch in *Streptococcus pyogenes* controls expression of a sodium: Alanine symporter family protein gene. Front Microbiol. 2018;9(200):1-10.

11 Lipfert J, Das R, Chu V B, Kudaravalli M, Boyd N, Herschlag D, Doniach S, Structural Transitions and Thermodynamics of a Glycine-Dependent Riboswitch from *Vibrio cholerae*. J Mol Biol. 2007;365(5):1393-406.

12 Kladwang W, Chou F C, Das R, Automated RNA Structure Prediction Uncovers a Kink-Turn Linker in Double Glycine Riboswitches. J Am Chem Soc. 2012;134(3):1404-7.
